# Associations Between Measures of Sarcopenic Obesity and Risk of Cardiovascular Disease and Mortality: A Cohort Study and Mendelian Randomization Analysis Using the UK Biobank

**DOI:** 10.1161/JAHA.118.011638

**Published:** 2019-06-21

**Authors:** Ruth E. Farmer, Rohini Mathur, A. Floriaan Schmidt, Krishnan Bhaskaran, Ghazaleh Fatemifar, Sophie V. Eastwood, Chris Finan, Spiros Denaxas, Liam Smeeth, Nish Chaturvedi

**Affiliations:** ^1^ Department of Non Communicable Disease Epidemiology London School of Hygiene & Tropical Medicine London United Kingdom; ^2^ Institute of Cardiovascular Science University College London United Kingdom; ^3^ The Institute of Computer Science University College London United Kingdom; ^4^ Division Heart and Lungs Department of Cardiology University Medical Center Utrecht Netherlands; ^5^ The Farr Institute of Health Informatics London United Kingdom; ^6^ The Institute of Health Informatics University College London London United Kingdom

**Keywords:** cardiovascular outcomes, epidemiology, grip strength, Mendelian randomization, obesity, Epidemiology, Obesity, Lifestyle, Risk Factors, Cardiovascular Disease

## Abstract

**Background:**

The “healthy obese” hypothesis suggests the risks associated with excess adiposity are reduced in those with higher muscle quality (mass/strength). Alternative possibilities include loss of muscle quality as people become unwell (reverse causality) or unmeasured confounding.

**Methods and Results:**

We conducted a cohort study using the UK Biobank (n=452 931). Baseline body mass index (BMI) was used to quantify adiposity and handgrip strength (HGS) used for muscle quality. Outcomes were fatal and non‐fatal cardiovascular disease, and mortality. As a secondary analysis we used waist‐hip‐ratio or fat mass percentage instead of BMI, and skeletal muscle mass index instead of HGS. In a subsample, we used gene scores for BMI, waist‐hip‐ratio and HGS in a Mendelian randomization (MR). BMI defined obesity was associated with an increased risk of all outcomes (hazard ratio [HR] range 1.10–1.82). Low HGS was associated with increased risks of cardiovascular and all‐cause mortality (HR range 1.39–1.72). HRs for the association between low HGS and cardiovascular disease events were smaller (HR range 1.05–1.09). There was no suggestion of an interaction between HGS and BMI to support the healthy obese hypothesis. Results using other adiposity metrics were similar. There was no evidence of an association between skeletal muscle mass index and any outcome. Factorial Mendelian randomization confirmed no evidence for an interaction. Low genetically predicted HGS was associated with an increased risk of mortality (HR range 1.08–1.19).

**Conclusions:**

Our analyses do not support the healthy obese concept, with no evidence that the adverse effect of obesity on outcomes was reduced by improved muscle quality. Lower HGS was associated with increased risks of mortality in both observational and MR analyses, suggesting reverse causality may not be the sole explanation.

**Clinical Perspective**

**What Is New?**

- In a large sample of UK adults, greater muscle quality, quantified by both directly measured and genetically predicted hand‐grip strength, was estimated to reduce cardiovascular and all‐cause mortality risk.
- There was no evidence for “healthy obesity:” higher body mass index was associated with higher cardiovascular and all‐cause mortality risk, and this was not attenuated in people with better muscle quality.

**What Are the Clinical Implications?**

- Grip strength may be a useful prognostic indicator for cardiovascular and all‐cause mortality risk, regardless of adiposity level.

The existence of a “healthy obese” phenotype, whereby a subgroup of individuals overcome the excess risk of cardiovascular disease (CVD) and overall mortality associated with obesity, remains debated. Proposed explanations for this paradox include: (1) a favorable metabolic profile, which has been widely studied^1^: and (2) the co‐occurrence of higher muscle mass or strength. Sarcopenia (the age‐related loss of muscle mass and strength), is also associated with adverse outcomes.^2^, ^3^, ^4^ Whether the effects of sarcopenia and obesity are cumulative, or whether an interaction exists is unclear. A recent review of prospective studies including 35 287 participants reported that individuals with both excess fat and sarcopenia (sarcopenic obesity) had a 24% higher risk of all‐cause mortality compared with individuals with sarcopenia alone or obesity alone, though confidence limits were wide (95% CI 12%–37%).^5^ The optimal measure of sarcopenia (muscle mass versus muscle quality) for event prediction remains contested.^6^ The optimal choice of obesity measure, whether central, (such as waist‐hip‐ratio [WHR]), or general (specifically body mass index [BMI]) is also unclear.^7^, ^8^, ^9^, ^10^, ^11^ Concerns of reverse causality between obesity and outcomes, particularly mortality, remain. For example, excess mortality in those who have both lost weight and developed sarcopenia as a consequence of disease may drive the appearance of a protective effect of obesity.

We utilized UK Biobank (UKB) to determine the association between sarcopenia, obesity and their interaction with CVD events and mortality. We examined effect modification by pre‐existing CVD and examined evidence for non‐linearity of these associations. As a secondary aim, we investigated the impact of using different metrics for adiposity and muscle quality. Mendelian randomization (MR) was used as a further casual inference method to explore the relationship between genetic instruments (and their interactions) for obesity and sarcopenia and CVD outcomes. Under certain assumptions, given that genetic variation is determined at conception, such associations are robust to both confounding and reverse causation.^12^

## Methods

### Study Population

The data that support the findings of this study are available to verified researchers upon application to the UKB in accordance with their access procedures (http://www.ukbiobank.ac.uk). The UKB cohort^13^, ^14^ recruited 502,641 men and women aged 40 and 69 years between 2006 and 2010 from primary care practices, spanning England, Scotland, and Wales. Participants underwent a baseline assessment capturing sociodemographic and lifestyle factors, and health status, including several measures of adiposity, muscle mass and muscle strength. Validated genotyping data were available from the initial release of genetic data for a random sample of 150 000 participants. White European participants from this sample with both phenotype and genotyping data passing quality control were eligible for MR analysis. UKB genotyping and imputation documentation are available at http://www.ukbiobank.ac.uk/scientists-3/genetic-data/. Longitudinal follow‐up for health‐related outcomes and mortality was available for all participants via linked secondary care records and death registrations via the office for national statistics.

This study had local approval from the UKB (project number 21893) and institutional approval from the London School of Hygiene & Tropical Medicine (application 15770). All participants provided informed consent at the time of recruitment to the UKB.

### Observed Phenotypes

#### Body mass index and grip strength

General obesity was defined using BMI, calculated with weight and height measurements taken at the baseline assessment. Muscle quality was defined as isometric handgrip strength (HGS), from the dominant hand using a Jamar dynamometer.^15^, ^16^, ^17^

We created binary variables for presence or absence of obesity or sarcopenia. The BMI cut‐off was >30 kg/m^2^. Sarcopenia was dichotomized using HGS cut‐offs at <30 kg for men and <20 kg for women.^18^

A composite of 4 mutually exclusive categories of body composition was generated. These were “optimal body composition” (ie, non‐obese and non‐sarcopenic), “obese non‐sarcopenic,” “sarcopenic non‐obese,” and “sarcopenic obesity.”

We further categorized each continuous exposure into quintiles to examine non‐linear associations between obesity and sarcopenia. The studied population equated to the following cut points: BMI: 23.55, 25.72, 27.85, and 30.82 kg/m^2^; HGS: 22, 28, 34, and 42 kg.

### Outcome Variables

Our primary outcomes of interest were first CVD event (fatal or non‐fatal) after cohort entry, CVD mortality and all‐cause mortality. CVD events were identified from linked hospital inpatient data using *International Classification of Diseases, Tenth Modification (ICD‐10)* codes (in any diagnostic position) of I00‐I99 (excluding I10–I15) and Q20 to Q28 (https://www.ucl.ac.uk/health‐informatics/caliber).^19^ Deaths were identified from linked death registration data if the relevant codes appeared anywhere in the record. Events were identified from baseline to March 31, 2015 (administrative end of complete linkage follow‐up).

### Covariates

Ethnicity was self‐reported at the nurse‐validated baseline assessment. Self‐report of diabetes mellitus status at baseline was adjudicated using the UKB algorithm.^20^ Deprivation was defined using the Townsend score, categorized into quintiles.^21^ Data on tobacco and alcohol consumption, and physical activity were obtained via the baseline touchscreen questionnaire. Smoking status was categorized into 6 groups: never smoker, ex‐smoker, 1 to 9, 10 to 19, 20 to 29, and ≥30 cigarettes per day. Frequency of alcohol intake at baseline was grouped into the following categories: never, special occasions only, once or twice a month, once or twice a week, and (almost) daily. Number of days a week where participants undertook >10 minutes of moderate physical activity and vigorous physical activity were treated as separate categorical variables. We chose not to include additional data on number of minutes per session to create composite variable for metabolic hours per week as this greatly reduced the sample size. History of CVD at baseline was ascertained using both baseline interview and linked secondary care data. Use of lipid lowering and anti‐hypertensive medications was reported and checked by a nurse, and blood pressure was measured. Data on high‐density lipoprotein, low‐density lipoprotein, and total cholesterol were not available.

### Statistical Analysis

Separate, cause‐specific Cox proportional hazard models (whereby deaths from other causes were censored), were fitted to estimate the association with each time to event outcome. Participants were considered at risk from 12 months after their baseline interview date to exclude events that could not be plausibly affected by baseline measures of body composition. In other words, we wanted to ensure that the results were not affected by reverse causality. This meant that participants experiencing the event of interest in the first 12 months were not included in the analysis. Follow‐up time either: (1) ended at the first occurrence of the outcome; or (2) was censored at the end of the follow‐up period or death (where death was not the outcome).

Risk of CVD events, CVD mortality, and all‐cause mortality was compared across the 4 categories of body composition, stratified by history of CVD at baseline, with “optimal body composition” as the reference category. All models were adjusted for the following known risk factors for CVD/mortality which are also likely to impact body composition/strength: age (linear term), sex, smoking status, ethnic group, deprivation, diabetes mellitus status, alcohol consumption, and moderate physical activity at baseline. Vigorous physical activity was omitted from the models, as it was not found to improve model fit or change any estimates of interest once moderate physical activity was included. We did not adjust for use of lipid lowering medications at baseline, as use of statins has been shown to not be a good proxy for current cholesterol and is more indicative of health seeking behavior and vascular risk;^22^ for which we could better adjust for using variables for smoking, alcohol, and physical activity. While blood pressure and cholesterol are strong predictors of CVD incidence, it is likely that these 2 variables are at least partly on the causal pathway between body composition and the outcomes or share common risk factors with our main exposures, making adjustment for them as “confounders” inappropriate and potentially misleading.^23^

Tests for interactions between BMI and HGS were performed using Wald tests.

**Figure 1:**
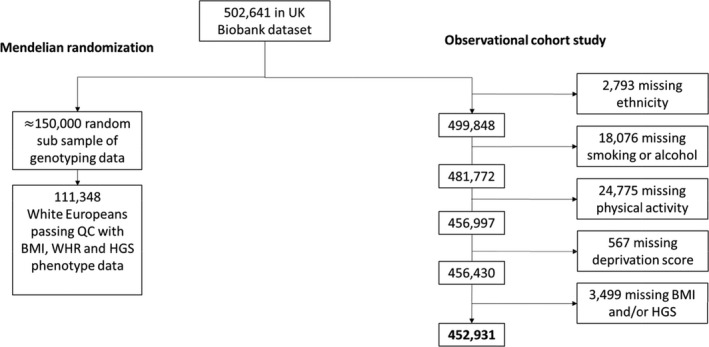
Flowchart of numbers included in study for observational analyses and Mendelian randomization. BMI indicates body mass index; HGS, hand‐grip strength; QC, quality control; WHR, waist‐hip‐ratio.

Participants with missing data for any model variable (<11%) were excluded (Figure 1). Missing values for covariates such as smoking, alcohol, and physical activity were likely missing not at random, so multiple imputation was not appropriate. We used a complete case analysis under the assumption that, conditional on model covariates, missingness was independent of the outcome.^24^

### Sensitivity Analysis

To further assess possible reverse causality, all analyses using the 4‐level variable for body composition were re‐run excluding the first 2, 3, then 4 years of follow‐up. Secondly, since it is possible that the inclusion of less severe CVD events in the composite CVD outcome may reduce any association or interaction, we also looked at coronary heart disease (CHD) (*ICD‐10* codes I20–25, I46, I47 and I49) as an additional outcome.

### Secondary Analyses

To investigate whether other measures of muscle quality or adiposity may provide differing results we repeated the primary analysis using skeletal muscle mass index (SMMI) in place of hand‐grip strength and replacing BMI with waist‐hip‐ratio (WHR) and then with fat mass (%). SMMI was calculated from the bioelectrical impedance measures using the Janssen et al equation, then taking the bottom 40% of the distribution as the definition of sarcopenia.^25^, ^26^, ^27^ The cut‐off for obesity for WHR was defined as ≥0.95 for men and ≥0.80 for women. Fat mass percentage does not have a well‐defined cut‐off in terms of obesity, therefore we used this metric to compare differences between quintiles only.

### Mendelian Randomization

After excluding potentially related samples, variants for BMI, WHR, and HGS, were screened on Hardy‐Weinberg equilibrium (threshold −log10 (*P* value) ≤1×10^−6^), and (where relevant) imputation quality (Table S1). We used previously identified independent variants to calculate weighted genetic instruments for BMI, WHR, and HGS.^28^, ^29^, ^30^, ^31^ Some of the identified variants did not pass quality control in our data and were therefore excluded from the genetic score (Tables S2 through S4). Weights were derived based on variant to phenotype associations. Two sets of weights were calculated, using internal and then external data^28^, ^29^, ^30^ to prevent weak instrument bias. Genetic score (GS) instrument strength was quantified using the F‐statistic of a linear regression model relating the GS to the intermediate phenotypes, with a statistic of ≥10 indicating the instrument had sufficient strength. Variants for HGS were taken from a previous study^29^ using the UKB as there are no published independent genome‐wide association studies. These weighted GS were subsequently associated with outcomes using Cox proportional hazard models, allowing for an interaction between BMI and HGS using a factorial MR (FMR) design.^32^ The FMR explored interactions by first categorizing the GS into high versus low (cut at the median), and combining these in a 4‐level categorical variable, and then by entering the 2 scores as continuous linear terms with an interaction. In line with the observational analysis, we then repeated this using WHR instead of BMI as a secondary analysis, and also considered CHD events and mortality in a sensitivity analysis. Interaction *P* values were obtained from likelihood ratio tests. To investigate pleiotropy, we also examined associations between individual variants used to develop the GS for each phenotype and other measured risk factors for CVD and mortality.

### Multiple Testing

Throughout our analyses, we made no adjustment for multiple testing. Provided results are appropriately interpreted, the adjustment in unnecessary, particularly when the multiple outcomes/exposures being tested are correlated as is the case here.^33^ We aimed to focus on the overall pattern of results, and used the CIs to provide the range of effect estimates for which our data were compatible rather than looking at whether a particular interval included or excluded the null. By the same argument, for the statistical tests of interaction, we also considered overall pattern of results rather than focusing on individual *P* values.

## Results

A total of 452 931 participants had complete data on BMI, HGS, and all covariates (Figure 1). Baseline characteristics stratified by body composition (according to BMI and HGS) are shown in Table 1. Twenty percent of participants had obesity but not sarcopenia, 11% had sarcopenia but not obesity, and 4% had both (sarcopenic obesity). Mean follow‐up time after excluding the first 12 months was 5.1 years. During follow‐up, there were 30 842 fatal and non‐fatal CVD events, and 11 336 deaths (3273 CVD).

**Table 1:** Baseline Characteristics of 452 931 Individuals in the UKB With BMI and Hand‐Grip Strength Measures and Complete Covariate Data, Stratified By Body Composition as Defined By BMI and Hand‐Grip Strength

Denominator	Optimal		Obese		Sarcopenic		Sarcopenic Obese	
	296 567 (65%)		89 906 (20%)		48 250 (11%)		18 208 (4%)	
Follow‐up time (y) (median, IQR)	6.18	5.48–6.86	6.17	5.46–6.88	6.00	5.31–6.67	6.01	5.32–6.72
Death during follow‐up, n (%)	6710	2.3%	2790	3.1%	1822	3.8%	827	4.5%
CVD death during follow up n (%)	1701	0.6%	991	1.1%	557	1.2%	308	1.7%
Any CVD event during follow‐up, n (%)	17 082	5.8%	8135	9.1%	3759	7.8%	2314	12.7%
Mean age at baseline (SD)	55.9	8.2	56.2	7.9	59.5	7.3	59.4	7.1
Male, n (%)	140 302	47.3%	45 979	51.1%	13 820	28.6%	5467	30.0%
Ethnic group, n (%)								
White	284 023	95.8%	85 297	94.9%	44 518	92.3%	16 826	92.4%
South Asian	3369	1.1%	869	1.0%	1657	3.4%	527	2.9%
African Caribbean	3495	1.2%	2237	2.5%	528	1.1%	420	2.3%
Other	5680	1.9%	1503	1.7%	1547	3.2%	435	2.4%
Townsend deprivation score (mean, SD)	−1.6	2.9	−1.0	3.2	−1.2	3.1	−0.4	3.4
Comorbidities, n (%)								
Type 1 diabetes mellitus	827	0.3%	335	0.4%	229	0.5%	111	0.6%
Type 2 diabetes mellitus	7149	2.4%	8786	9.8%	2241	4.6%	2813	15.4%
History of CHD	11 599	3.9%	7061	7.9%	3077	6.4%	2146	11.8%
History of CVD (CHD, stroke, angina)	33 226	11.2%	15 140	16.8%	7585	15.7%	4321	23.7%
Smoking status, n (%)								
Never smoker	208 804	70.4%	57 030	63.4%	34 242	71.0%	11 914	65.4%
Ex‐smoker	65 794	22.2%	27 014	30.0%	10 341	21.4%	5144	28.3%
Current‐smoker	21 969	7.4%	5862	6.5%	3667	7.6%	1150	6.3%
1 to 9 cigarettes per day	4760	1.6%	920	1.0%	790	1.6%	171	0.9%
10 to 19 cigarettes per day	9407	3.2%	2347	2.6%	1681	3.5%	473	2.6%
20 to 29 cigarettes per day	6274	2.1%	1920	2.1%	952	2.0%	378	2.1%
30+ cigarettes per day	1528	0.5%	675	0.8%	244	0.5%	128	0.7%
Alcohol consumption, n (%)								
Never	18 696	6.3%	7650	8.5%	5638	11.7%	2895	15.9%
Special occasions only	27 608	9.3%	12 837	14.3%	6814	14.1%	3828	21.0%
Once or twice a month	30 881	10.4%	12 209	13.6%	5255	10.9%	2438	13.4%
Once or twice a week	76 936	25.9%	24 131	26.8%	12 034	24.9%	4312	23.7%
3 or 4 times a week	75 610	25.5%	18 285	20.3%	9637	20.0%	2637	14.5%
Almost daily	66 836	22.5%	14 794	16.5%	8872	18.4%	2098	11.5%
PA (median [IQR])								
Days of moderate PA per week	4	[2,6]	3	[1,5]	4	[2,6]	3	[1,5]
Days of vigorous PA per week	2	[0,3]	1	[0,3]	1	[0,3]	0	[0,2]
Anthropometric/metabolic measures (mean, SD)								
Systolic blood pressure	136.47	18.62	142.01	17.69	136.50	19.27	141.33	18.64
BMI, kg/m^2^	25.32	2.69	33.81	3.74	25.19	2.82	34.37	4.74
Whole body fat mass (%)	28.80	7.51	37.62	7.75	31.83	7.31	41.18	6.98
Waist‐hip‐ratio	0.85	0.08	0.92	0.09	0.85	0.08	0.91	0.09
Skeletal muscle mass index, kg/m^2^	7.63	1.39	8.65	1.50	6.96	1.30	7.98	1.49
Grip strength, kg	34.14	10.02	34.97	10.54	17.74	5.63	17.35	11.29

BMI indicates body mass index; CHD, coronary heart disease; CVD, cardiovascular disease; IQR, interquartile range; PA, physical activity.

### Observational Analysis

**Figure 2:**
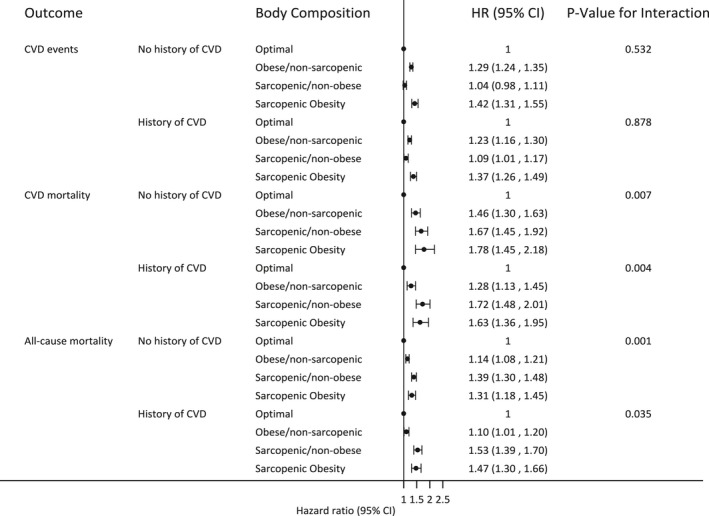
Estimated association between body composition and fatal/non‐cardiovascular disease (CVD) events and cause‐specific and all‐cause mortality. Obesity measured as body mass index >30, sarcopenia measured as HGS <30 kg men and <20 kg women.

In participants with no history of CVD, BMI‐defined obesity in the absence of sarcopenia was associated with an increased risk of CVD events (adjusted hazard ratio [HR], 1.29 [1.24–1.35]). The analogous estimate for those with prior CVD was 1.23 (1.16–1.30) (Figure 2). Obesity in the absence of sarcopenia was also associated with an increased risk of mortality (Figure 2).

Sarcopenia without obesity had a more modest association with risk of CVD events, relative to optimal body composition, regardless of prior CVD (HR range, 1.04–1.09). Estimated associations with mortality were larger (HR range, 1.39–1.72) (Figure 2).

Sarcopenic obesity (defined by HGS and BMI) was associated with the highest risk of CVD events compared with the other 3 categories of body composition, with no clear differences between those with and without prior CVD. For mortality outcomes, sarcopenic obesity individuals had estimated risks consistent with sarcopenic non‐obese individuals, again with minimal differences by CVD history (Figure 2).

There was no clear evidence of an interaction between sarcopenia and obesity for CVD events. The relative effects of obesity appeared similar with and without the presence of sarcopenia, and vice versa, with and without prior CVD (Figure 2). Statistically, there was evidence of interactions between sarcopenia and obesity for mortality outcomes, but the direction of this was suggestive of a reduced relative effect of obesity in those who were sarcopenic. Further, the estimated size of the interactions was not large in magnitude (Figure 2).

Risk of CVD events increased linearly by quintile of BMI, but within BMI quintile, there was no clear pattern of an association with HGS (Tables 2 and 3). In contrast, for the mortality outcomes, there was less evidence of an association with BMI quintile, but there was clear pattern of decreasing risk with increasing HGS quintile. A “U” shaped association was observed for the effect of BMI on CVD mortality and all‐cause mortality in the lowest quintile of HGS, particularly for those with a prior history of CVD. In general, however, there was little evidence of non‐linearity (Tables 2 and 3). There was no other strong suggestion that the effect of BMI differed by HGS, or vice versa, as reflected in both the estimates of effect and interaction *P* values.

**Table 2:** Adjusted^a^ Hazard Ratios (with 95% CI) of All‐Cause and CVD Mortality, and Combined Fatal/Non‐CVD Events By Quintiles of BMI and HGS, in Patients With No Prior History of CVD

	HGS Quintile (kg)					
	43 to 90	35 to 42	29 to 34	23 to 28	0 to 22	*P* Value for Interaction
BMI quintile (kg/m^2^)						
All‐cause mortality						
12 to 23	1 (ref)	1.37 (1.12–1.69)	1.52 (1.23–1.87)	1.84 (1.50–2.26)	2.23 (1.82–2.73)	0.169
23 to 26	0.92 (0.75–1.15)	1.17 (0.96–1.44)	1.63 (1.33–2.00)	1.70 (1.38–2.09)	1.88 (1.53–2.31)	
26 to 28	1.02 (0.83–1.24)	1.17 (0.96–1.42)	1.40 (1.14–1.72)	1.62 (1.31–2.00)	1.92 (1.56–2.37)	
28 to 31	1.14 (0.94–1.39)	1.23 (1.01–1.49)	1.51 (1.23–1.85)	1.67 (1.35–2.06)	2.05 (1.67–2.52)	
31 to 60	1.35 (1.10–1.65)	1.39 (1.14–1.70)	1.67 (1.36–2.05)	1.83 (1.49–2.26)	2.07 (1.69–2.53)	
Fatal/non‐fatal CVD						
12 to 23	1 (ref)	0.92 (0.77–1.09)	1.06 (0.90–1.25)	1.03 (0.87–1.22)	0.96 (0.81–1.14)	0.376
23 to 26	0.98 (0.83–1.15)	1.03 (0.88–1.21)	1.06 (0.90–1.25)	1.06 (0.90–1.26)	1.08 (0.92–1.28)	
26 to 28	1.11 (0.95–1.29)	1.13 (0.96–1.31)	1.10 (0.93–1.30)	1.14 (0.96–1.35)	1.11 (0.94–1.32)	
28 to 31	1.15 (0.98–1.34)	1.19 (1.02–1.39)	1.22 (1.04–1.44)	1.18 (0.99–1.40)	1.34 (1.13–1.58)	
31 to 60	1.51 (1.30–1.76)	1.31 (1.12–1.54)	1.60 (1.37–1.87)	1.48 (1.25–1.74)	1.49 (1.27–1.75)	
Fatal CVD						
12 to 23	1 (ref)	1.38 (0.90–2.12)	1.39 (0.88–2.18)	1.80 (1.14–2.83)	2.63 (1.71–4.06)	0.225
23 to 26	0.94 (0.60–1.47)	1.20 (0.79–1.83)	1.52 (0.98–2.35)	2.32 (1.50–3.59)	2.38 (1.53–3.70)	
26 to 28	1.07 (0.71–1.64)	1.15 (0.76–1.74)	1.32 (0.85–2.05)	1.54 (0.96–2.46)	2.12 (1.35–3.34)	
28 to 31	1.30 (0.87–1.96)	1.35 (0.90–2.03)	1.72 (1.13–2.63)	1.91 (1.22–3.00)	3.06 (2.00–4.70)	
31 to 60	1.63 (1.08–2.45)	1.69 (1.13–2.54)	2.54 (1.69–3.82)	2.89 (1.89–4.42)	2.55 (1.66–3.92)	

BMI indicates body mass index; CVD, cardiovascular disease; HGS, hand‐grip strength.

a Adjusted for age, sex, ethnicity, and baseline measures of smoking, alcohol consumption, diabetes mellitus status, physical activity and deprivation.

**Table 3:** Adjusted^a^ Hazard Ratios (with 95% CI) of All‐Cause and CVD Mortality, and Combined Fatal/Non‐CVD Events By Quintiles of BMI and HGS, in Patients With Prior History of CVD

	HGS Quintile (kg)					
	43 to 90	35 to 42	29 to 34	23 to 28	0 to 22	*P* Value for Interaction
BMI quintile (kg/m^2^)						
All‐cause mortality						
12 to 23	1 (ref)	1.49 (0.95–2.32)	2.03 (1.31–3.13)	2.95 (1.92–4.53)	3.45 (2.27–5.24)	0.007
23 to 26	1.31 (0.84–2.04)	1.33 (0.87–2.03)	1.86 (1.22–2.85)	2.10 (1.35–3.25)	2.15 (1.39–3.32)	
26 to 28	1.23 (0.81–1.89)	1.30 (0.86–1.96)	1.93 (1.28–2.92)	1.88 (1.21–2.91)	2.01 (1.31–3.09)	
28 to 31	1.21 (0.80–1.83)	1.44 (0.96–2.16)	1.50 (0.99–2.26)	1.63 (1.05–2.51)	2.01 (1.32–3.07)	
31 to 60	1.45 (0.96–2.18)	1.54 (1.03–2.29)	1.58 (1.05–2.37)	2.31 (1.53–3.48)	2.74 (1.82–4.10)	
Fatal/non‐fatal CVD						
12 to 23	1 (ref)	1.06 (0.80–1.39)	1.26 (0.96–1.65)	1.14 (0.86–1.5)	1.25 (0.96–1.63)	0.070
23 to 26	1.11 (0.85–1.45)	1.33 (1.03–1.70)	1.29 (1.00–1.68)	1.19 (0.90–1.56)	1.26 (0.97–1.64)	
26 to 28	1.29 (1.00–1.65)	1.31 (1.03–1.68)	1.29 (1.00–1.67)	1.36 (1.05–1.78)	1.30 (1.00–1.69)	
28 to 31	1.24 (0.97–1.58)	1.32 (1.04–1.69)	1.21 (0.94–1.56)	1.3 (1.00–1.69)	1.47 (1.14–1.89)	
31 to 60	1.66 (1.30–2.11)	1.42 (1.12–1.80)	1.68 (1.32–2.14)	1.59 (1.24–2.04)	1.79 (1.41–2.29)	
Fatal CVD						
12 to 23	1 (ref)	2.34 (1.02–5.33)	3.40 (1.51–7.65)	4.69 (2.07–10.61)	6.76 (3.06–14.93)	0.146
23 to 26	1.82 (0.79–4.18)	2.45 (1.11–5.39)	2.64 (1.18–5.93)	3.87 (1.71–8.74)	4.12 (1.82–9.29)	
26 to 28	2.03 (0.91–4.51)	2.37 (1.09–5.15)	3.53 (1.62–7.69)	3.57 (1.59–8.02)	3.09 (1.36–7.02)	
28 to 31	1.57 (0.71–3.49)	2.65 (1.23–5.71)	2.66 (1.22–5.81)	3.35 (1.51–7.45)	3.97 (1.79–8.77)	
31 to 60	2.85 (1.32–6.16)	2.96 (1.38–6.34)	3.31 (1.54–7.13)	4.26 (1.96–9.26)	5.21 (2.41–11.28)	

BMI indicates body mass index; CVD; cardiovascular disease; HGS, hand‐grip strength.

a Adjusted for age, sex, ethnicity, and baseline measures of smoking, alcohol consumption, diabetes mellitus status, physical activity and deprivation.

### Sensitivity Analyses

Extending the exclusion period at the beginning of follow‐up appeared to make minimal differences to the results (Figure S1). Results for CHD events and mortality had similar pattern to those for CVD events and mortality as a whole, though in general effect sizes for the effect of obesity were larger in magnitude (Figure S2 and Tables S5 and S6).

### Secondary Analyses

The proportion of individuals with sarcopenic obesity at baseline differed substantially for each of the 4 definitions; varying from 2% using BMI and SMMI, to 17% using WHR and SSMI (Table S7). Associations between sarcopenia as defined by SMMI and all outcomes were generally null compared with those with normal body composition (Figure S3). Further, there was no consistent evidence of an association between SMMI quintiles and any outcome, regardless of BMI quintile (Tables S8 and S9).

Using WHR instead of BMI gave similar results for all analyses performed (Figure S4 and Tables S10 and S11). Using fat mass quintiles gave a similar pattern of results as BMI, though there was stronger evidence for an interaction with HGS. However, this appeared to be driven by the same U‐shaped association observed for BMI at the lower HGS quintiles. (Tables S12 and S13).

### MR Analysis

**Figure 3:**
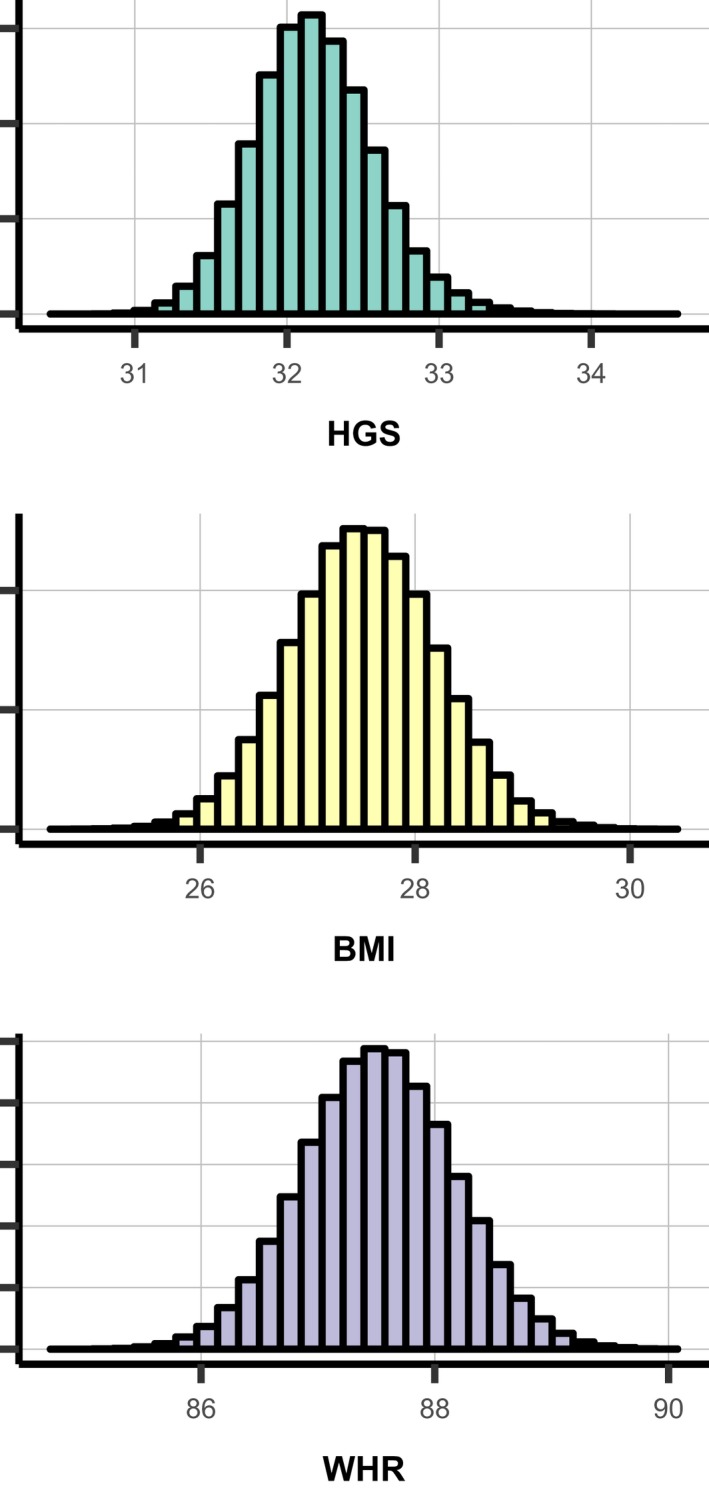
Distributions of genetic risk scores for hand‐grip strength (HGS) (kg) (top), BMI (kg/m^2^) (middle) and waist‐hip‐ratio (WHR) (bottom). BMI indicates body mass index; HGS, hand‐grip strength; WHR, waist‐hip‐ratio.

A total of 111 348 participants contributed to MR (Figure 1). The median genetically predicted values for BMI, WHR, and HGS were 27.5 kg/m^2^, 0.88, and 32.2 kg, respectively (Figure 3). The F statistics for the 3 scores were 2009, 500, and 128 using external weights; and 2250, 569, and 137 with internal weights for BMI, WHR, and HGS, respectively, indicating that both internal and external genetic scores (GS) were strong instruments.

**Figure 4:**
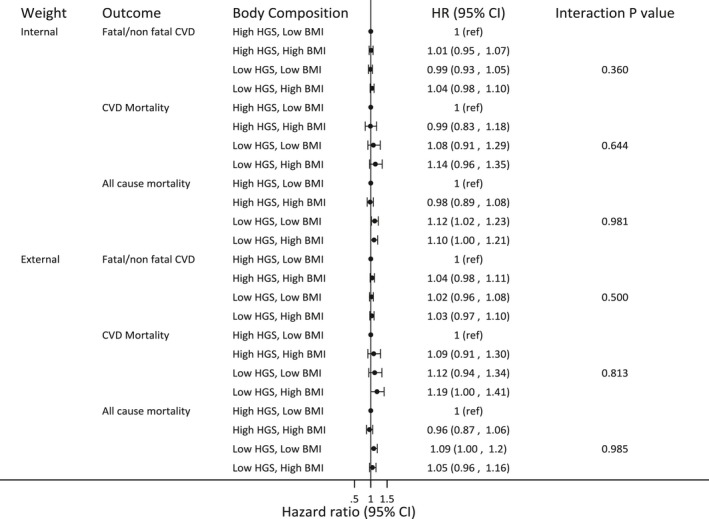
Relative hazard of fatal/non‐fatal cardiovascular disease (CVD) events and cause specific and all‐cause mortality according to category of body composition as defined by genetic scores estimated from both external in internal weights in a factorial Mendelian randomization analysis.

The factorial analysis suggested no statistical evidence of interaction between BMI and HGS (Figure 4). In general, there was no clear evidence of an increased risk of fatal/non‐fatal CVD for any combination of genetic scores for BMI and HGS. All HRs were close to 1 for both internal and externally weighted GS (maximum HR: 1.04, minimum HR: 0.99). Broadly, low HGS was estimated to increase the risk of mortality outcomes compared with high HGS, irrespective of obesity category (Figure 4), though the effect sizes were smaller than those from the observational analysis. In participants with high HGS scores, there was no evidence of any effect of BMI on CVD and all‐cause mortality (HRs 1.09 [0.91–1.30] and 0.96 [0.87–1.06], respectively for externally weighted GS, and 0.99 [0.83–1.18] and 0.98 [0.89–1.08] for internally weighted GS. This lack of BMI effect was also observed in those with low HGS (Figure 4).

Using internally weighted GS as continuous variables instead of dichotomizing at the median, there remained no evidence for an interaction between BMI and HGS for any of the outcomes (Table 4). As with the dichotomized risk scores, the estimated HRs per unit increase in BMI for all outcomes from these models were estimated to small in magnitude (HR range, 1.00–1.04) and all CIs included 1. This was also true for the effect of grip strength on fatal/non‐fatal CVD (HR 1.02 [0.97–1.08]). However, each unit increase in HGS was estimated to reduce the relative risk of all‐cause and CVD mortality by around 10% at the median BMI score (HR 0.86 [0.79–0.94] and 0.88 [0.75–1.02], respectively).

**Table 4:** Results of MR Using Continuous Genetic Scores^a^ With Interaction Between HGS and BMI

Outcome	HR Per Unit Increase in HGS At the Median BMI	HR Per Unit Increase in BMI at the Median HGS	Interaction Term (HR)	Interaction *P* Value
Fatal/non‐fatal CVD	1.02 (0.97–1.08)	1.04 (1.00–1.07)	0.97 (0.89–1.05)	0.434
CVD mortality	0.88 (0.75–1.02)	1.03 (0.94–1.13)	1.12 (0.89–1.40)	0.344
All‐cause mortality	0.86 (0.79–0.94)	1.00 (0.96–1.06)	1.08 (0.95–1.22)	0.239

BMI indicates body mass index; CVD, cardiovascular disease; HGS, hand‐grip strength; HR, hazard ratio; WHR, waist‐hip‐ratio.

a Genetic risk score derived from internal weights.

Results using genetic scores for WHR instead of BMI were similar (Figure S5 and Table S14), as were results for CHD and CHD mortality (Figure S6 and Table S14).

### Assessment of Potential Pleiotropy

**Figure 5:**
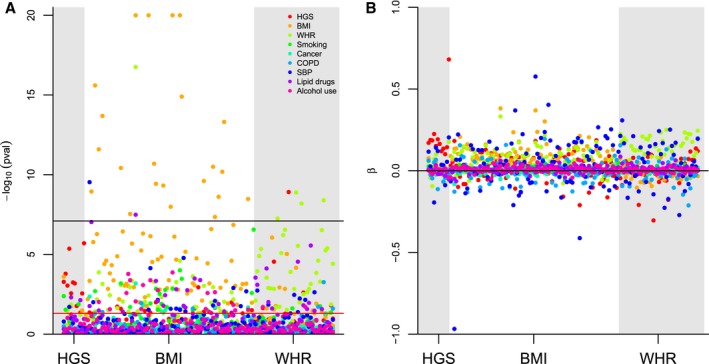
Associations between HGS (left) BMI (middle) and WHR (right) variants, and possible pleiotropic variables. BMI indicates body mass index; COPD, chronic obstructive pulmonary disease; HGS, hand‐grip strength; WHR, waist‐hip‐ratio; SBP, systolic blood pressure.

Three BMI variants were identified that were genome‐wide significantly associated with systolic blood pressure (SBP), WHR, and use of lipid‐lowering drugs (Figure 5A). One WHR SNP was found to also be associated with HGS. Although not reaching genome‐wide statistical significance, many SNPs had relatively large beta estimates for an association with SBP (Figure 5B).

## Discussion

In a study of 450 000 UK adults, we found no evidence for a healthy obesity phenotype in association with muscle strength using observed phenotypes in a multivariable regression or using Mendelian randomization approaches in 111 348 individuals with available genetic and phenotype data. Increased BMI was linearly associated with increased risk of both CVD events and mortality (CVD and all‐cause) to a similar degree. Greater muscle quality, as measured by HGS or by variants acting as instrumental variables were estimated to reduced CVD and all‐cause mortality risk but showed less association (both statistically and in magnitude) with fatal/non‐fatal CVD events. Our overall findings do not support a “U” or “J” shaped association between adiposity and any outcome; however, few participants were clinically underweight. There was no consistent evidence that the effect of adiposity was modified by grip strength or skeletal muscle mass, and vice‐versa, suggesting that the negative effects of obesity are not reduced in those with greater strength, or that the observed reduction in risk of mortality with increasing strength is not negated by excess adiposity.

Previous studies that have examined the joint effect of grip strength and obesity are limited by small sample size and potential for reverse causality.^34^, ^35^ For the latter issue, our findings were unchanged when we excluded events occurring in the first and subsequent 2, 3, and 4 years of follow‐up, and when stratified by prior history of CVD. UKB participants are relatively young; relatively small numbers have pre‐existing disease or extreme anthropometric phenotypes,^36^ which could explain this. Indeed, the proportion of clinically underweight, at <1%, and obese, at 4%, was low, also making it harder to detect non‐linear associations if they exist.

From secondary analyses, our finding of similarity between BMI and WHR in terms of subsequent risk is in line with a recent comparative study of 4 UK‐based cohorts.^11^ The finding that muscle strength defined by HGS appears to be of greater prognostic value than muscle mass defined by SMMI adds to a growing body of evidence;.^5^, ^37^

In contrast to a recent study also using the UKB,^4^ we found no clear evidence of an effect of sarcopenia on incident or recurring CVD events. However, the existing study used a narrower definition of CVD which may explain the stronger effects observed.

In our observational analysis, we found that sarcopenia was strongly related to mortality, consistent with existing observational evidence.^3^, ^4^ Despite similar findings in those with and without prior CVD suggesting that reverse causality had little impact on findings, we cannot exclude the possibility of some misclassification of prior CVD because of undiagnosed disease at baseline. However, the number of undiagnosed individuals would have to be large to explain this fully. Further, in contrast to a previous, smaller MR study,^29^ the harmful effect of lower genetically predicted HGS on mortality outcomes was replicated when using genetically predicted HGS, most notably for all‐cause mortality. This suggests it is even less likely that the adverse effect of sarcopenia on mortality is solely attributable to reverse causality.

Potential causal mechanisms for the observed association between HGS and mortality outcomes include the effect of skeletal muscle on exercise capacity, itself a strong predictor of mortality, via maintenance of preload and cardiac output during physical activity.^38^

The MR analysis also found no evidence of an interaction between adiposity and HGS. In our observational analysis, there was some suggestion of a protective effect of obesity in sarcopenic individuals for mortality outcomes, which resulted in some statistical evidence of interactions. This direction of interaction is not consistent with the healthy obese hypothesis and may be a consequence of un‐measured confounding by frailty.

A key strength of the study was the additional use of MR analyses, an important tool in establishing causality. However, the following issues deserve consideration. Given the sparsity of GWAS data on HGS, SNP selection^29^ as well as estimation utilized the same UKB sample, resulting in a degree of selection bias (eg, inflated weights). This type of selection bias decreases to zero as sample size increase.^39^ Given the size of the UKB we expect this source of bias to have minimal influence on presented results. Our results are also strengthened by the finding that using external weights for BMI, WHR, and HGS showed similar results, and the F statistics for both internal and external weights were large for all predicted phenotypes.

An alternative explanation for our findings is that genes that predict grip strength may also predict other phenotypes associated with health status (pleiotropic effects). Because of the focus on factorial MR, we were unable to use more robust methods such as MR‐egger that protect against bias because of horizontal pleiotropy and acknowledge this may be a limitation of our study. However, we were able to explore the pleiotropy potential of the used variants by examining the associations between the genetic risk scores and other CVD risk factors. Here, we found only 3 genome‐wide significant associations with potentially pleiotropic pathways such as SBP and use of lipid‐lowering drugs. However, these pathways may well lie on the causal pathway between our exposures (BMI, WHR, and HGS) and CVD, which does not bias or invalidate our results (horizontal pleiotropy). Having said this, there may exist alternative pleiotropic pathways that we were unable to examine given the available data.

As expected, distributions of the genetic risk scores for BMI, WHR, and HGS showed far less variation than the observed phenotypes. Genetically predicted HGS ranged from 30 to 35 kg, BMI ranged from 27 to 31. This may explain the lack of effect of observed genetic associations and small HR estimates for BMI and WHR on any of the outcomes when dichotomized at the median genetic score. This could also contribute to the lack of interaction observed here. Further, we cannot exclude the possibility that we were underpowered to detect the interactions in this analysis given the smaller effect sizes, though given our sample size we believe this unlikely.

Our study is limited by mean follow‐up period of 5 years, limiting the number of incident events available for analysis. Indeed, the number of deaths occurring in our sample (11 336) was comparable with the number of deaths observed in the 2016 meta‐analysis (14 306), which had a far smaller overall sample size. However, the previously mentioned healthy participant bias may explain the overall lower event rates and limited our ability to look at people with severe obesity but should not bias associations between obesity and outcomes.

A further limitation is that the lack of repeated measures of obesity and sarcopenia prevent us from examining the role of prolonged exposure or changes in exposure, which may be important predictors of our outcomes of interest. Other risk factors for our outcomes may also have been time varying, making choice of covariates for confounder adjustment complex. Since the data available for both exposure and covariates were all measured at a single time point, our analysis relies on making assumptions about which covariates are likely to have influenced the exposure at that point, versus which may have been a consequence of the measured exposure (so should not be adjusted for). Provided the assumptions of MR were satisfied, our analysis using genetic risk scores is robust to both of these limitations of the observational analysis.

## Conclusions

Our analyses do not support the concept of a “healthy obese” phenotype in relationship to muscle mass or grip strength. WHR, BMI, and fat mass defined obesity appear similar in their association with future cardiovascular risk and mortality, in that in general, increased adiposity increases risk. Lower‐grip strength was associated with increased risks of cardiovascular and all‐cause mortality in a multivariable regression and MR analysis, suggesting reverse causality may not be the sole explanation. Grip strength appears to be a good prognostic indicator for mortality risk in adults with and without obesity, though it is not yet clear whether interventions to improve muscle strength would directly decrease risk.

## Sources of Funding

This work was funded by a Diabetes UK/British Heart Foundation award to Prof. Chaturvedi and Prof. Smeeth, award number [15/0005250]. Dr. Mathur is supported by a Sir Henry Wellcome Postdoctoral Fellowship from the Wellcome Trust (WT/201375/Z/16/Z). Prof. Bhaskaran holds a Sir Henry Dale fellowship jointly funded by the Wellcome Trust and the Royal Society (107731/Z/15/Z).

## Disclosures

None.

## Supporting information


**Table S1.** Genetic Quality Control (QC) details
**Table S2.** Waist Hip Ratio SNPs Identified From Previous Study, and Internal/External Weights Used for MR Analyses
**Table S3.** BMI SNPs and Internal/External Weights Used for MR Analyses
**Table S4.** Hand‐Grip Strength SNPs and Internal/External Weights Used for MR Analyses
**Table S5**. Adjusted* Hazard (with 95% confidence interval) Ratios of Combined Fatal/Non‐CHD Events and Mortality By Quintiles of BMI and HGS for Those Without History of CVD
**Table S6.** Adjusted* Hazard (with 95% confidence interval) Ratios of Combined Fatal/Non‐CHD Events and Mortality By Quintiles of BMI and HGS for Those With History of CVD
**Table S7.** Distribution of Obesity and Sarcopenia At Baseline By Sex
**Table S8.** Adjusted* Hazard Ratios (with 95% confidence interval) of All‐Cause and CVD Mortality and Combined Fatal/Non‐CVD Events By Quintiles of BMI and SMMI in Those Without With a History of CV
**Table S9.** Adjusted* Hazard Ratios (with 95% confidence interval) of All‐Cause and CVD Mortality and Combined Fatal/Non‐CVD Events By Quintiles of BMI and SMMI in Those With a History of CVD
**Table S10.** Adjusted* Hazard Ratios (with 95% confidence interval) of All‐Cause and CVD Mortality and Combined Fatal/Non‐CVD Events By Quintiles of WHR and HGS in Participants Without History of CVD
**Table S11.** Adjusted* Hazard Ratios (with 95% confidence interval) of All‐Cause and CVD Mortality and Combined Fatal/Non‐CVD Events By Quintiles of WHR and HGS in Participants With History of CVD
**Table S12.** Adjusted* Hazard Ratios (with 95% confidence interval) of All‐Cause and CVD Mortality and Combined Fatal/Non‐CVD Events By Quintiles of Fat Mass (%) and HGS in Participants Without History of CVD
**Table S13.** Adjusted* Hazard Ratios (with 95% confidence interval) of All‐Cause and CVD Mortality, and Combined Fatal/Non‐CVD Events By Quintiles of Fat Mass (%) and HGS in Participants With History of CVD
**Table S14.** Results of MR Using Continuous Genetic Risk Scores With Interaction Between HGS and BMI/WHR
**Figure S1.** Estimated association between body composition and fatal/non‐fatal CHD and CVD events and cause‐specific and all‐cause mortality excluding the first 2, 3, and 4 years of follow‐up: BMI and hand‐grip strength.
**Figure S2.** Estimated association between body composition and fatal/non‐CHD events and mortality. Obesity measured as BMI >30, sarcopenia measured as HGS <30 kg men and <20 kg women.
**Figure S3.** Estimated association between body composition and fatal/non‐CVD events and cause‐specific and all‐cause mortality. Obesity measured as BMI >30, sarcopenia measured as SMMI in bottom 40%.
**Figure S4.** Estimated association between body composition and fatal/non‐CVD events and cause‐specific and all‐cause mortality. Obesity measured as WHR ≥0.95 in men and ≥0.80 in women, sarcopenia measured as HGS <30 kg men and <20 kg women.
**Figure S5.** Relative hazard of fatal/non‐fatal CHD and CVD events and cause‐specific and all‐cause mortality according to category of body composition (using WHR) as defined by genetic scores in a factorial Mendelian randomization analysis, where weights for the genetic score were determined from both external and internal data.
**Figure S6.** Relative hazard of fatal/non‐fatal CHD events and mortality according to category of body composition as defined by genetic scores estimated from both external in internal weights in a factorial Mendelian randomization analysis.
