# Supplementary material for: Associations Between Measures of Sarcopenic Obesity and Risk of Cardiovascular Disease and Mortality: A Cohort Study and Mendelian Randomization Analysis Using the UK Biobank
Source: J Am Heart Assoc. 2019 Jun 21;8(13):e011638. doi: 10.1161/JAHA.118.011638 (PMC6662360; doi:10.1161/JAHA.118.011638)

SUPPLEMENTAL MATERIAL

Table S1. Genetic Quality Control (QC) details.

Level	QC operation	Notes
**Individual**	Sex mismatch	Drop patients with a mismatch between genetic sex and self-reported sex at baseline
**Individual**	Recommended UK BeLIVE exclusions	Recommended by UK Biobank for the 50,005 patients genotyped using UK BeLIVE.
**Individual**	Recommend exclusion	Recommended genomic exclusion for poor heterozygosity/missingness
**Individual**	Genetic relatedness	Kinship coefficient 0.044 – remove all related individuals
**Individual**	Population origin	Drop non-CEU participants
**Individual**	Heterozygosity	Filter out samples with heterozygosity (as taken from the "heterozygosity" column of the sample file, which must be present) outside the interval [0.18,0.2]
**Individual**	Sample-missing-rate	Filter out samples with missing data rate (as taken from the "missing" column of the sample file) greater than 5%
**SNP**[‡]	Information score	Filter out SNPs with Fisher information < 0.3
**SNP**	SNP missing call rate	Filter out SNPs with missing call rate greater than or equal 5%
**SNP**	Hardy-Weinberg Equilibrium	HWE threshold -log10(p-value) <= $1x10^{-6}$
**SNP**	Minor Allele Frequency	MAF outside 0.01-1 range
**SNP**	SNP missing rate	Filter out SNPs with missing data rate greater than or equal to 5%

Table S2. Waist hip ratio SNPs identified from previous study, and internal/external weights used for MR analyses.

Waist Hip Ratio					
Rsid	Effect Allele	MAF internal	Internal Weight	MAF external	External Weight
Included in Genetic Risk Score					
rs10195252	T	0.594	0.122	0.558	0.027
rs10245353	A	0.176	0.206	0.183	0.035
rs1045241	C	0.742	0.058	0.692	0.019
rs10804591	A	0.790	0.138	0.850	0.024
rs10842707	T	0.208	0.179	0.167	0.032
rs10919388	C	0.761	0.196	0.717	0.024
rs10991437	A	0.117	0.248	0.100	0.031
rs11231693	A	0.049	0.226	0.042	0.041
rs12454712	T	0.624	0.053	0.633	0.016
rs12608504	A	0.365	0.127	0.342	0.022
rs12679556	G	0.235	0.108	0.208	0.027
rs1294410	C	0.622	0.171	0.625	0.031
rs1358980	T	0.460	0.197	0.450	0.039
rs1385167	G	0.123	0.078	0.142	0.029
rs1440372	C	0.751	0.068	0.742	0.024
rs1443512	A	0.217	0.200	0.200	0.028
rs1534696	C	0.461	0.165	0.350	0.011
rs1569135	A	0.552	0.234	0.533	0.021
rs1776897	G	0.089	0.232	0.075	0.03
rs17819328	G	0.418	0.184	0.450	0.021
rs1936805	T	0.519	0.226	0.550	0.042
rs224333	G	0.642	0.011	0.667	0.02
rs2276824	C	0.439	0.099	0.483	0.024
rs2294239	A	0.572	0.146	0.550	0.025
rs2371767	G	0.738	0.248	0.792	0.036
rs2645294	T	0.571	0.209	0.535	0.031
rs2820443	T	0.704	0.214	0.700	0.035
rs2925979	T	0.301	0.149	0.283	0.018
rs303084	A	0.794	0.150	0.783	0.023
rs3805389	A	0.243	0.049	0.242	0.012
rs4081724	G	0.870	0.126	0.850	0.035
rs4646404	G	0.670	0.129	0.625	0.027
rs4765219	C	0.667	0.185	0.625	0.028
rs6556301	T	0.357	0.146	0.375	0.022
rs714515	G	0.436	0.193	0.458	0.027
rs7705502	A	0.298	0.037	0.292	0.027
rs7830933	A	0.776	0.091	0.742	0.022
rs7917772	A	0.632	0.046	0.683	0.014
rs8030605	A	0.124	0.004	0.158	0.03
rs8066985	A	0.485	0.126	0.517	0.018
rs905938	T	0.733	0.101	0.675	0.025
rs9687846	A	0.201	0.167	0.192	0.024
rs979012	T	0.365	0.085	0.358	0.027

rs9991328	T	0.458	0.113	0.483	0.018
Excluded SNPs that did not pass the overall QC in our data processing step					
rs17451107					
rs6090583					
rs7759742					
rs7801581					
rs8042543					

Table S3. BMI SNPs and internal/external weights used for MR analyses.

BMI					
Rsid	Effect Allele	MAF internal	Internal Weight	MAF external	External Weight
Included in Genetic Risk Score					
rs1000940	G	0.30	0.060	0.225	0.019
rs10132280	C	0.72	0.085	0.667	0.023
rs1016287	T	0.30	0.102	0.325	0.023
rs10182181	G	0.49	0.159	0.500	0.031
rs10938397	G	0.43	0.134	0.433	0.040
rs10968576	G	0.32	0.119	0.292	0.025
rs11030104	A	0.80	0.204	0.800	0.041
rs11057405	G	0.89	0.132	0.908	0.031
rs11126666	A	0.25	0.004	0.308	0.021
rs11165643	T	0.60	0.073	0.575	0.022
rs11191560	C	0.08	0.147	0.058	0.031
rs11583200	C	0.40	0.091	0.375	0.018
rs1167827	G	0.57	0.111	0.542	0.020
rs11688816	G	0.56	0.060	0.458	0.017
rs11727676	T	0.90	0.020	0.925	0.036
rs11847697	T	0.04	0.057	0.042	0.049
rs12286929	G	0.52	0.035	0.433	0.022
rs12401738	A	0.37	0.065	0.425	0.021
rs12429545	A	0.12	0.124	0.100	0.033
rs12446632	G	0.86	0.107	0.867	0.040
rs12566985	G	0.43	0.052	0.425	0.024
rs12885454	C	0.65	0.077	0.633	0.021
rs12940622	G	0.56	0.093	0.542	0.018
rs13021737	G	0.84	0.295	0.875	0.060
rs13078960	G	0.20	0.097	0.183	0.030
rs13107325	T	0.07	0.209	0.117	0.048
rs13191362	A	0.88	0.130	0.800	0.028
rs13201877	G	0.10	0.038	0.083	0.023
rs1441264	A	0.61	0.079	0.550	0.018
rs1460676	C	0.15	0.079	0.217	0.020
rs1516725	C	0.87	0.158	0.908	0.045
rs1528435	T	0.63	0.072	0.583	0.018
rs1558902	A	0.40	0.384	0.450	0.082
rs16851483	T	0.07	0.141	0.092	0.048
rs16907751	C	0.92	0.116	0.958	0.035
rs16951275	T	0.77	0.163	0.775	0.031
rs17024393	C	0.02	0.384	0.042	0.066
rs17094222	C	0.18	0.075	0.208	0.025
rs17203016	G	0.18	0.080	0.200	0.021
rs17405819	T	0.70	0.073	0.633	0.022
rs17724992	A	0.76	0.112	0.692	0.019
rs1808579	C	0.51	0.104	0.525	0.017

rs1928295	T	0.57	0.051	0.575	0.019
rs2033732	C	0.74	0.016	0.758	0.019
rs205262	G	0.26	0.143	0.267	0.022
rs2080454	C	0.39	0.068	0.392	0.017
rs2112347	T	0.64	0.121	0.625	0.026
rs2121279	T	0.12	0.017	0.117	0.025
rs2176040	A	0.35	0.043	0.392	0.014
rs2176598	T	0.25	0.117	0.200	0.020
rs2207139	G	0.17	0.203	0.100	0.045
rs2287019	C	0.84	0.180	0.850	0.036
rs2365389	C	0.59	0.135	0.658	0.020
rs2650492	A	0.27	0.001	0.308	0.021
rs2820292	C	0.57	0.101	0.508	0.020
rs2836754	C	0.64	0.062	0.650	0.016
rs29941	G	0.67	0.090	0.667	0.018
rs3101336	C	0.60	0.129	0.649	0.033
rs3736485	A	0.46	0.057	0.425	0.018
rs3810291	A	0.68	0.135	0.625	0.028
rs3817334	T	0.41	0.143	0.450	0.026
rs3849570	A	0.32	0.032	0.367	0.019
rs3888190	A	0.41	0.111	0.358	0.031
rs4256980	G	0.66	0.103	0.725	0.021
rs4740619	T	0.55	0.067	0.533	0.018
rs4787491	G	0.55	0.095	0.614	0.016
rs492400	C	0.43	0.016	0.325	0.016
rs543874	G	0.21	0.237	0.267	0.048
rs6091540	C	0.73	0.094	0.725	0.019
rs6465468	T	0.27	0.005	0.325	0.017
rs6477694	C	0.35	0.025	0.358	0.017
rs6567160	C	0.23	0.235	0.283	0.056
rs657452	A	0.40	0.002	0.417	0.023
rs6804842	G	0.56	0.058	0.575	0.019
rs7138803	A	0.37	0.162	0.442	0.032
rs7141420	T	0.52	0.091	0.617	0.024
rs7164727	T	0.67	0.108	0.775	0.018
rs7239883	G	0.38	0.063	0.317	0.016
rs7243357	T	0.84	0.066	0.867	0.022
rs758747	T	0.25	0.079	0.267	0.023
rs7599312	G	0.76	0.093	0.708	0.022
rs7715256	G	0.43	0.072	0.450	0.016
rs7899106	G	0.05	0.090	0.050	0.040
rs7903146	C	0.71	0.130	0.750	0.023
rs9374842	T	0.78	0.057	0.742	0.019
rs9400239	C	0.71	0.082	0.700	0.019
rs9540493	A	0.47	0.068	0.450	0.017
rs9641123	C	0.40	0.054	0.392	0.019
rs977747	T	0.42	0.087	0.467	0.017
rs9914578	G	0.20	0.030	0.167	0.020

Excluded SNPs that did not pass the overall QC in our data processing step					
rs10733682					
rs12016871					
rs17001654					
rs2033529					
rs2075650					
rs9925964					

Table S4. Hand grip strength SNPs and internal/external weights used for MR analyses.

Hand Grip Strength					
Rsid	Effect Allele	MAF internal	Internal Weight	MAF external	External Weight
Included in Genetic Risk Score					
rs10186876	A	0.353	0.102	0.360	0.113
rs10861798	A	0.429	0.225	0.430	0.159
rs2110927	C	0.261	0.130	0.270	0.098
rs2273555	A	0.607	0.169	0.610	0.096
rs2288278	A	0.658	0.150	0.660	0.147
rs374532236	T	0.357	0.185	0.380	0.121
rs4926611	C	0.643	0.117	0.640	0.115
rs6565586	A	0.243	0.190	0.250	0.096
rs6687430	G	0.459	0.166	0.460	0.125
rs72762373	A	0.019	0.681	0.030	0.359
rs72979233	A	0.756	0.185	0.760	0.112
rs958685	A	0.516	0.143	0.520	0.164
Excluded SNPs that did not pass the overall QC in our data processing step					
rs11614333					
rs34845616					
rs78325334					
rs80103986					

Table S5. Adjusted* hazard (with 95% confidence interval) ratios of combined fatal/non CHD events and mortality by quintiles of BMI and HGS for those without history of CVD.

		**Fatal/non-fatal CHD**					
		**HGS Quintile (kg)**					
		**43-90**	**35-42**	**29-34**	**23-28**	**0-22**	**P value for interaction**
**BMI Quintile (kg/m2)**	**12-23**	1 (ref)	0.84 (0.59 , 1.19)	1.01 (0.71 , 1.43)	1.02 (0.71 , 1.47)	1.64 (1.18 , 2.29)	0.101
	**23-26**	1.15 (0.84 , 1.57)	1.28 (0.94 , 1.73)	1.34 (0.97 , 1.85)	1.78 (1.28 , 2.48)	1.57 (1.12 , 2.21)	
	**26-28**	1.47 (1.09 , 1.97)	1.51 (1.12 , 2.02)	1.7 (1.25 , 2.31)	1.69 (1.21 , 2.36)	1.89 (1.36 , 2.64)	
	**28-31**	1.47 (1.1 , 1.98)	1.6 (1.2 , 2.15)	1.81 (1.33 , 2.46)	1.88 (1.35 , 2.61)	2.21 (1.59 , 3.05)	
	**31-60**	2.32 (1.74 , 3.09)	1.76 (1.31 , 2.37)	2.41 (1.78 , 3.26)	2.36 (1.72 , 3.25)	2.59 (1.89 , 3.54)	
		**CHD Mortality**					
		**HGS Quintile (kg)**					
		**43-90**	**35-42**	**29-34**	**23-28**	**0-22**	**P value for interaction**
	**12-23**	1 (ref)	0.94 (0.5 , 1.8)	1.18 (0.6 , 2.32)	1.49 (0.72 , 3.08)	3.44 (1.82 , 6.51)	0.342
	**23-26**	0.97 (0.52 , 1.8)	1.13 (0.63 , 2.04)	1.4 (0.74 , 2.64)	2.72 (1.44 , 5.12)	3.09 (1.61 , 5.93)	
	**26-28**	1.17 (0.66 , 2.08)	1.38 (0.78 , 2.43)	1.4 (0.75 , 2.61)	1.44 (0.7 , 2.97)	3.64 (1.93 , 6.87)	
	**28-31**	1.4 (0.8 , 2.46)	1.33 (0.75 , 2.34)	1.73 (0.95 , 3.15)	2.6 (1.39 , 4.86)	3.12 (1.64 , 5.94)	
	**31-60**	1.75 (1 , 3.08)	1.77 (1.01 , 3.11)	2.49 (1.4 , 4.43)	2.7 (1.43 , 5.08)	2.73 (1.44 , 5.17)	

*Adjusted for age, sex, ethnicity and baseline measures of smoking, alcohol consumption, diabetes status physical activity and deprivation. BMI – body mass index HGS – hand grip strength

Table S6. Adjusted* hazard (with 95% confidence interval) ratios of combined fatal/non CHD events and mortality by quintiles of BMI and HGS for those with history of CVD.

		**Fatal/non-fatal CHD**					
		**HGS Quintile (kg)**					
		**43-90**	**35-42**	**29-34**	**23-28**	**0-22**	**P value for interaction**
**BMI Quintile (kg/m2)**	**12-23**	1 (ref)	1.24 (0.82 , 1.87)	1.16 (0.76 , 1.77)	0.97 (0.62 , 1.52)	1.46 (0.98 , 2.19)	0.455
	**23-26**	1.45 (0.98 , 2.15)	1.77 (1.22 , 2.58)	1.88 (1.28 , 2.76)	1.41 (0.93 , 2.13)	1.73 (1.16 , 2.57)	
	**26-28**	1.78 (1.23 , 2.59)	1.8 (1.25 , 2.6)	1.8 (1.24 , 2.62)	1.98 (1.34 , 2.93)	2 (1.36 , 2.94)	
	**28-31**	1.84 (1.28 , 2.65)	1.92 (1.34 , 2.76)	1.68 (1.16 , 2.45)	1.76 (1.2 , 2.6)	2.18 (1.49 , 3.18)	
	**31-60**	2.47 (1.73 , 3.54)	2.24 (1.57 , 3.21)	2.29 (1.59 , 3.29)	2.3 (1.58 , 3.33)	2.81 (1.95 , 4.05)	
		**CHD Mortality**					
		**HGS Quintile (kg)**					
		**43-90**	**35-42**	**29-34**	**23-28**	**0-22**	**P value for interaction**
	**12-23**	1 (ref)	1.83 (0.6 , 5.56)	2.32 (0.77 , 7)	4.32 (1.45 , 12.84)	4.23 (1.44 , 12.49)	0.745
	**23-26**	2.07 (0.7 , 6.09)	2.28 (0.81 , 6.45)	2.84 (0.99 , 8.19)	2.66 (0.88 , 8.07)	3.98 (1.35 , 11.73)	
	**26-28**	2.03 (0.71 , 5.78)	2.09 (0.75 , 5.83)	3.05 (1.09 , 8.55)	4.05 (1.41 , 11.62)	3.06 (1.04 , 9)	
	**28-31**	1.62 (0.57 , 4.6)	2.45 (0.89 , 6.74)	2.68 (0.96 , 7.45)	2.8 (0.97 , 8.06)	2.81 (0.97 , 8.15)	
	**31-60**	2.49 (0.9 , 6.87)	2.99 (1.1 , 8.15)	3.17 (1.15 , 8.7)	3.46 (1.24 , 9.67)	4.21 (1.51 , 11.69)	

*Adjusted for age, sex, ethnicity and baseline measures of smoking, alcohol consumption, diabetes status physical activity and deprivation**.** BMI – body mass index HGS – hand grip strength

Table S7. Distribution of Obesity and Sarcopenia at baseline by sex.

	Overall		Male		Female	
BMI and grip Strength	N	%	N	%	N	%
Non-Obese/Non-Sarcopenic	296,567	65.5%	140,302	68.3%	156,265	63.2%
Obese	89,906	19.8%	45,979	22.4%	43,927	17.8%
Sarcopenic	48,250	10.7%	13,820	6.7%	34,430	13.9%
Sarcopenic Obesity	18,208	4.0%	5,467	2.7%	12,741	5.2%
BMI and SMMI	N	%	N	%	N	%
Non-Obese/Non-Sarcopenic	170,532	38.1%	75,449	37.2%	95,083	38.9%
Obese	96,354	21.5%	45,824	22.6%	50,530	20.6%
Sarcopenic	170,428	38.1%	76,764	37.8%	93,664	38.3%
Sarcopenic Obesity	10,247	2.3%	4,790	2.4%	5,457	2.2%
WHR and grip Strength	N	%	N	%	N	%
Non-Obese/Non-Sarcopenic	206,533	45.5%	115,795	56.2%	90,738	36.6%
Obese	182,592	40.2%	71,634	34.8%	110,958	44.8%
Sarcopenic	23,714	5.2%	8,402	4.1%	15,312	6.2%
Sarcopenic Obesity	40,842	9.0%	10,187	4.9%	30,655	12.4%
WHR and SMMI	N	%	N	%	N	%
Non-Obese/Non-Sarcopenic	125,213	28.0%	68,397	33.7%	56,816	23.2%
Obese	144,608	32.3%	54,738	27.0%	89,870	36.7%
Sarcopenic	102,360	22.9%	54,227	26.7%	48,133	19.7%
Sarcopenic Obesity	75,308	16.8%	25,420	12.5%	49,888	20.4%

WHR – waist hip ratio, BMI – body mass index HGS – hand grip strength SMMI – skeletal muscle mass index

Table S8. Adjusted* hazard (with 95% confidence interval) ratios of all-cause and CVD mortality, and combined fatal/non CVD events by quintiles of BMI and SMMI in those without with a history of CVD.

BMI Quintile (kg/m2)						
**All-cause mortality**						
	**SMMI Quintile**					
	**9.2-31.2**	**8.3-9.2**	**7.0-8.3**	**6.3-7.0**	**4.2-6.3**	**P value for interaction**
**12-23**	1 (ref)	0.8 (0.58 , 1.1)	1 (0.73 , 1.36)	1.02 (0.73 , 1.43)	0.87 (0.63 , 1.21)	0.061
**23-26**	0.80 (0.57 , 1.13)	0.74 (0.55 , 1.01)	0.90 (0.66 , 1.23)	0.80 (0.57 , 1.12)	0.86 (0.62 , 1.19)	
**26-28**	0.72 (0.52 , 0.99)	0.76 (0.56 , 1.03)	0.96 (0.69 , 1.32)	0.82 (0.58 , 1.15)	0.81 (0.58 , 1.14)	
**28-31**	0.84 (0.62 , 1.13)	0.86 (0.63 , 1.17)	0.92 (0.66 , 1.28)	0.79 (0.56 , 1.11)	0.95 (0.67 , 1.34)	
**31-60**	0.97 (0.71 , 1.30)	0.99 (0.72 , 1.36)	0.92 (0.66 , 1.27)	0.88 (0.62 , 1.24)	0.97 (0.65 , 1.44)	
**Fatal/non-fatal CVD**						
	**SMMI Quintile**					
	**9.2-31.2**	**8.3-9.2**	**7.0-8.3**	**6.3-7.0**	**4.2-6.3**	**P value for interaction**
**12-23**	1 (ref)	1.28 (0.92 , 1.78)	1.36 (0.98 , 1.88)	1.31 (0.93 , 1.84)	1.20 (0.86 , 1.68)	0.004
**23-26**	1.43 (1.02 , 2.01)	1.35 (0.98 , 1.87)	1.19 (0.86 , 1.65)	1.28 (0.91 , 1.81)	1.42 (1.02 , 1.99)	
**26-28**	1.38 (1.00 , 1.91)	1.47 (1.07 , 2.02)	1.68 (1.21 , 2.34)	1.32 (0.94 , 1.86)	1.37 (0.97 , 1.92)	
**28-31**	1.60 (1.17 , 2.20)	1.51 (1.09 , 2.08)	1.65 (1.18 , 2.31)	1.47 (1.04 , 2.06)	1.54 (1.09 , 2.19)	
**31-60**	2.00 (1.46 , 2.74)	1.92 (1.38 , 2.66)	1.83 (1.32 , 2.56)	1.61 (1.14 , 2.27)	1.93 (1.32 , 2.82)	
**Fatal CVD**						
	**SMMI Quintile**					
	**9.2-31.2**	**8.3-9.2**	**7.0-8.3**	**6.3-7.0**	**4.2-6.3**	**P value for interaction**
**12-23**	1 (ref)	1.49 (0.64 , 3.45)	1.85 (0.81 , 4.23)	1.77 (0.73 , 4.34)	1.37 (0.57 , 3.27)	0.297
**23-26**	1.44 (0.6 , 3.45)	1.43 (0.63 , 3.25)	1.56 (0.68 , 3.6)	1.43 (0.58 , 3.53)	1.86 (0.78 , 4.47)	
**26-28**	1.27 (0.55 , 2.93)	1.4 (0.62 , 3.19)	1.61 (0.69 , 3.77)	1.32 (0.53 , 3.25)	1.32 (0.54 , 3.27)	
**28-31**	1.78 (0.79 , 4.02)	1.62 (0.71 , 3.68)	2.1 (0.89 , 4.96)	1.37 (0.56 , 3.37)	2.22 (0.9 , 5.45)	
**31-60**	2.34 (1.04 , 5.27)	1.91 (0.82 , 4.44)	2.32 (0.98 , 5.5)	1.4 (0.56 , 3.5)	1.68 (0.6 , 4.71)	

*Adjusted for age, sex, ethnicity and baseline measures of smoking, alcohol consumption, diabetes status physical activity and deprivation BMI- body mass index. SMMI – skeletal muscle mass index

Table S9. Adjusted* hazard (with 95% confidence interval) ratios of all-cause and CVD mortality, and combined fatal/non CVD events by quintiles of BMI and SMMI in those with with a history of CVD.

BMI Quintile (kg/m2)	All-cause mortality					
	SMMI Quintile					
	**9.2-31.2**	**8.3-9.2**	**7.0-8.3**	**6.3-7.0**	**4.2-6.3**	**P value for interaction**
**12-23**	1 (ref)	0.66 (0.36 , 1.24)	0.93 (0.51 , 1.68)	1.24 (0.65 , 2.36)	1.06 (0.57 , 1.97)	0.012
**23-26**	0.83 (0.44 , 1.56)	0.69 (0.38 , 1.25)	0.85 (0.47 , 1.54)	0.72 (0.37 , 1.4)	0.65 (0.34 , 1.23)	
**26-28**	0.69 (0.38 , 1.25)	0.65 (0.36 , 1.17)	0.91 (0.5 , 1.65)	0.72 (0.37 , 1.38)	0.65 (0.34 , 1.25)	
**28-31**	0.65 (0.36 , 1.16)	0.77 (0.43 , 1.37)	0.63 (0.34 , 1.17)	0.7 (0.37 , 1.33)	0.54 (0.27 , 1.07)	
**31-60**	0.79 (0.45 , 1.41)	0.9 (0.5 , 1.61)	0.9 (0.49 , 1.67)	0.79 (0.42 , 1.5)	0.76 (0.36 , 1.57)	
	**Fatal/non-fatal CVD**					
	**SMMI Quintile**					
	**9.2-31.2**	**8.3-9.2**	**7.0-8.3**	**6.3-7.0**	**4.2-6.3**	**P value for interaction**
**12-23**	1 (ref)	0.79 (0.49 , 1.27)	0.93 (0.59 , 1.48)	0.89 (0.54 , 1.46)	0.93 (0.58 , 1.48)	0.281
**23-26**	1.08 (0.67 , 1.75)	0.9 (0.57 , 1.41)	1.12 (0.71 , 1.76)	0.97 (0.6 , 1.58)	0.96 (0.59 , 1.54)	
**26-28**	1.06 (0.67 , 1.68)	1.02 (0.65 , 1.59)	1.17 (0.74 , 1.84)	0.84 (0.52 , 1.36)	1.02 (0.63 , 1.64)	
**28-31**	1.02 (0.66 , 1.6)	1.04 (0.67 , 1.63)	1.17 (0.74 , 1.87)	1.02 (0.63 , 1.64)	0.94 (0.58 , 1.54)	
**31-60**	1.37 (0.88 , 2.13)	1.13 (0.72 , 1.78)	1.23 (0.77 , 1.96)	1.17 (0.73 , 1.88)	1.09 (0.64 , 1.84)	
	**Fatal CVD**					
	**SMMI Quintile**					
	**9.2-31.2**	**8.3-9.2**	**7.0-8.3**	**6.3-7.0**	**4.2-6.3**	**P value for interaction**
**12-23**	1 (ref)	0.34 (0.16 , 0.74)	0.53 (0.26 , 1.07)	0.42 (0.17 , 1.02)	0.53 (0.24 , 1.14)	0.05
**23-26**	0.43 (0.2 , 0.96)	0.40 (0.20 , 0.80)	0.5 (0.25 , 1.02)	0.36 (0.15 , 0.86)	0.27 (0.11 , 0.63)	
**26-28**	0.42 (0.2 , 0.85)	0.40 (0.20 , 0.8)0	0.55 (0.27 , 1.13)	0.3 (0.12 , 0.72)	0.32 (0.14 , 0.74)	
**28-31**	0.4 (0.2 , 0.79)	0.45 (0.23 , 0.89)	0.41 (0.19 , 0.86)	0.39 (0.17 , 0.89)	0.2 (0.08 , 0.53)	
**31-60**	0.58 (0.29 , 1.12)	0.59 (0.30 , 1.18)	0.4 (0.19 , 0.86)	0.4 (0.18 , 0.9)	0.3 (0.1 , 0.88)	

*Adjusted for age, sex, ethnicity and baseline measures of smoking, alcohol consumption, diabetes status physical activity and deprivation BMI- body mass index. SMMI – skeletal muscle mass index

Table S10. Adjusted* hazard (with 95% confidence interval) ratios of all-cause and CVD mortality, and combined fatal/non CVD events by quintiles of WHR and HGS in participants without history of CVD.

WHR Quintile	43-90	35-42	29-34	23-28	0-22	P value for interaction
**All-cause mortality**						
**HGS Quintile (kg)**						
**0.44-0.79**	1 (ref)	1.01 (0.56 , 1.83)	1.02 (0.59 , 1.76)	1.18 (0.69 , 2.02)	1.29 (0.76 , 2.21)	0.138
**0.79-0.85**	0.66 (0.37 , 1.17)	0.71 (0.40 , 1.24)	1.10 (0.64 , 1.90)	1.18 (0.69 , 2.02)	1.48 (0.87 , 2.53)	
**0.85-0.90**	0.77 (0.45 , 1.32)	0.90 (0.53 , 1.54)	1.11 (0.65 , 1.91)	1.22 (0.71 , 2.09)	1.47 (0.86 , 2.51)	
**0.90-0.95**	0.78 (0.46 , 1.33)	1.03 (0.60 , 1.75)	1.17 (0.68 , 1.99)	1.33 (0.77 , 2.28)	1.63 (0.95 , 2.80)	
**0.95-1.30**	1.13 (0.67 , 1.92)	1.17 (0.69 , 2.00)	1.47 (0.86 , 2.49)	1.70 (0.99 , 2.90)	1.77 (1.03 , 3.05)	
**Fatal/non-fatal CVD**						
**HGS Quintile (kg)**	**43-90**	**35-42**	**29-34**	**23-28**	**0-22**	**P value for interaction**
**0.44-0.79**	1 (ref)	1.33 (0.79 , 2.23)	1.24 (0.76 , 2.02)	1.17 (0.72 , 1.90)	1.18 (0.72 , 1.92)	0.500
**0.79-0.85**	1.42 (0.86 , 2.34)	1.32 (0.80 , 2.16)	1.45 (0.89 , 2.36)	1.41 (0.87 , 2.29)	1.37 (0.84 , 2.23)	
**0.85-0.90**	1.34 (0.83 , 2.18)	1.29 (0.79 , 2.09)	1.46 (0.90 , 2.38)	1.49 (0.92 , 2.43)	1.52 (0.93 , 2.47)	
**0.90-0.95**	1.55 (0.96 , 2.50)	1.60 (0.99 , 2.59)	1.56 (0.96 , 2.54)	1.47 (0.90 , 2.40)	1.63 (1.00 , 2.66)	
**0.95-1.30**	1.86 (1.15 , 3.00)	1.69 (1.04 , 2.72)	1.89 (1.16 , 3.06)	1.87 (1.15 , 3.05)	1.88 (1.15 , 3.07)	
**Fatal CVD**						
**HGS Quintile (kg)**	**43-90**	**35-42**	**29-34**	**23-28**	**0-22**	**P value for interaction**
**0.44-0.79**	1 (ref)	1.41 (0.40 , 5.02)	0.76 (0.22 , 2.57)	1.06 (0.33 , 3.44)	1.13 (0.35 , 3.65)	0.418
**0.79-0.85**	0.81 (0.24 , 2.75)	0.68 (0.20 , 2.32)	1.21 (0.37 , 3.95)	1.29 (0.40 , 4.17)	1.83 (0.57 , 5.84)	
**0.85-0.90**	0.83 (0.26 , 2.64)	0.83 (0.26 , 2.66)	1.12 (0.35 , 3.60)	1.22 (0.38 , 3.93)	1.69 (0.53 , 5.42)	
**0.90-0.95**	0.84 (0.26 , 2.64)	1.14 (0.36 , 3.58)	1.22 (0.38 , 3.87)	1.74 (0.54 , 5.57)	1.92 (0.60 , 6.17)	
**0.95-1.30**	1.30 (0.41 , 4.07)	1.31 (0.42 , 4.10)	1.78 (0.57 , 5.58)	2.16 (0.68 , 6.85)	2.32 (0.73 , 7.39)	

*Adjusted for age, sex, ethnicity and baseline measures of smoking, alcohol consumption, diabetes status physical activity and deprivation. WHR- waist hip ratio. HGS – hand grip strength

Table S11. Adjusted* hazard (with 95% confidence interval) ratios of all-cause and CVD mortality, and combined fatal/non CVD events by quintiles of WHR and HGS in participants with history of CVD.

WHR Quintile	43-90	35-42	29-34	23-28	0-22	P value for interaction
**All-cause mortality**						
**HGS Quintile (kg)**						
**0.44-0.79**	1 (ref)	1.73 (0.38 , 7.91)	1.26 (0.3 , 5.34)	1.63 (0.4 , 6.73)	1.6 (0.39 , 6.55)	0.911
**0.79-0.85**	0.98 (0.23 , 4.28)	1.35 (0.32 , 5.64)	1.33 (0.32 , 5.49)	1.77 (0.43 , 7.22)	2.14 (0.53 , 8.68)	
**0.85-0.90**	1.09 (0.27 , 4.45)	1.23 (0.3 , 5.01)	1.6 (0.39 , 6.51)	1.86 (0.45 , 7.58)	2.05 (0.51 , 8.34)	
**0.90-0.95**	1.1 (0.27 , 4.47)	1.26 (0.31 , 5.1)	1.7 (0.42 , 6.88)	1.89 (0.47 , 7.68)	2.31 (0.57 , 9.35)	
**0.95-1.30**	1.49 (0.37 , 5.99)	1.59 (0.39 , 6.38)	1.81 (0.45 , 7.27)	2.28 (0.56 , 9.2)	2.75 (0.68 , 11.1)	
**Fatal/non-fatal CVD**						
**HGS Quintile (kg)**						
	**43-90**	**35-42**	**29-34**	**23-28**	**0-22**	**P value for interaction**
**0.44-0.79**	1 (ref)	1.15 (0.51 , 2.62)	0.85 (0.39 , 1.83)	0.94 (0.44 , 2)	1.04 (0.49 , 2.21)	0.706
**0.79-0.85**	1.33 (0.61 , 2.9)	0.98 (0.45 , 2.13)	1.18 (0.55 , 2.52)	1.09 (0.51 , 2.32)	1.11 (0.52 , 2.35)	
**0.85-0.90**	1.08 (0.51 , 2.3)	1.11 (0.52 , 2.36)	1.19 (0.56 , 2.54)	1.15 (0.54 , 2.44)	1.21 (0.57 , 2.57)	
**0.90-0.95**	1.11 (0.53 , 2.34)	1.21 (0.57 , 2.55)	1.22 (0.58 , 2.58)	1.18 (0.55 , 2.51)	1.32 (0.62 , 2.81)	
**0.95-1.30**	1.34 (0.63 , 2.81)	1.27 (0.6 , 2.68)	1.33 (0.63 , 2.81)	1.33 (0.63 , 2.81)	1.46 (0.69 , 3.09)	
**Fatal CVD**						
**HGS Quintile (kg)**						
	**43-90**	**35-42**	**29-34**	**23-28**	**0-22**	**P value for interaction**
**0.44-0.79**	1 (ref)	2.03 (0.24 , 17.5)	0.81 (0.1 , 6.79)	1.07 (0.14 , 8.2)	1.35 (0.18 ,10.03)	0.418
**0.79-0.85**	0.35 (0.04 , 3.41)	1.15 (0.15 , 8.8)	1.42 (0.19 ,10.68)	1.76 (0.24 ,12.99)	1.83 (0.25 ,13.38)	
**0.85-0.90**	0.81 (0.11 , 6.04)	1.09 (0.15 , 7.97)	1.39 (0.19 ,10.21)	1.66 (0.22 ,12.22)	1.53 (0.21 ,11.18)	
**0.90-0.95**	0.79 (0.11 , 5.75)	1.16 (0.16 , 8.37)	1.36 (0.19 , 9.83)	1.49 (0.2 , 10.91)	2.04 (0.28 ,14.81)	
**0.95-1.30**	1.24 (0.17 , 8.89)	1.43 (0.2 , 10.26)	1.65 (0.23 ,11.87)	2.14 (0.3 , 15.4)	2.61 (0.36 ,18.76)	

*Adjusted for age, sex, ethnicity and baseline measures of smoking, alcohol consumption, diabetes status physical activity and deprivation. WHR- waist hip ratio. HGS – hand grip strength

Table S12. Adjusted* hazard (with 95% confidence interval) ratios of all-cause and CVD mortality, and combined fatal/non CVD events by quintiles of Fat mass (%) and HGS in participants without history of CVD.

Fat mass quintile (%)	43-90	35-42	29-34	23-28	0-22	P value for interaction
**All-cause mortality**						
**HGS Quintile (kg)**						
**5-23.8**	1 (ref)	1.39 (1.23 , 1.58)	1.82 (1.57 , 2.10)	2.21 (1.84 , 2.65)	3.12 (2.57 , 3.78)	<0.001
**23.9-28.6**	1.23 (1.08 , 1.41)	1.34 (1.17 , 1.52)	1.55 (1.34 , 1.80)	1.87 (1.58 , 2.23)	2.64 (2.21 , 3.16)	
**28.7-33.5**	1.42 (1.23 , 1.65)	1.49 (1.29 , 1.71)	1.77 (1.53 , 2.06)	2.00 (1.70 , 2.35)	2.10 (1.78 , 2.48)	
**33.6-39.2**	1.61 (1.29 , 2.01)	1.65 (1.37 , 1.99)	1.82 (1.54 , 2.15)	2.02 (1.73 , 2.36)	2.22 (1.90 , 2.59)	
**39.3-69.8**	2.89 (1.88 , 4.45)	1.58 (1.17 , 2.14)	2.04 (1.70 , 2.44)	2.05 (1.75 , 2.41)	2.52 (2.16 , 2.93)	
**Fatal/non-fatal CVD**						
**HGS Quintile (kg)**						
	**43-90**	**35-42**	**29-34**	**23-28**	**0-22**	**P value for interaction**
**5-23.8**	1 (ref)	1.02 (0.93 , 1.13)	1.05 (0.93 , 1.19)	1.22 (1.03 , 1.43)	1.41 (1.17 , 1.70)	0.002
**23.9-28.6**	1.23 (1.12 , 1.36)	1.09 (0.99 , 1.21)	1.22 (1.09 , 1.37)	1.11 (0.95 , 1.29)	1.21 (1.02 , 1.43)	
**28.7-33.5**	1.26 (1.13 , 1.41)	1.24 (1.12 , 1.39)	1.25 (1.11 , 1.41)	1.31 (1.15 , 1.49)	1.22 (1.06 , 1.41)	
**33.6-39.2**	1.64 (1.39 , 1.94)	1.46 (1.26 , 1.68)	1.51 (1.33 , 1.72)	1.45 (1.28 , 1.65)	1.30 (1.14 , 1.48)	
**39.3-69.8**	1.44 (0.93 , 2.25)	1.64 (1.32 , 2.03)	1.78 (1.55 , 2.04)	1.60 (1.41 , 1.81)	1.77 (1.57 , 2.00)	
**Fatal CVD**						
**HGS Quintile (kg)**						
	**43-90**	**35-42**	**29-34**	**23-28**	**0-22**	**P value for interaction**
**5-23.8**	1 (ref)	1.32 (1.02 , 1.72)	1.64 (1.21 , 2.22)	2.84 (2 , 4.04)	3.67 (2.48 , 5.42)	0.050
**23.9-28.6**	1.41 (1.08 , 1.83)	1.38 (1.06 , 1.81)	1.68 (1.25 , 2.26)	2.20 (1.54 , 3.13)	2.86 (1.93 , 4.22)	
**28.7-33.5**	1.59 (1.18 , 2.13)	1.64 (1.24 , 2.17)	1.99 (1.47 , 2.7)	2.24 (1.58 , 3.17)	2.59 (1.81 , 3.71)	
**33.6-39.2**	2.02 (1.34 , 3.05)	2.12 (1.49 , 3.02)	2.38 (1.69 , 3.37)	2.42 (1.71 , 3.44)	2.82 (2.02 , 3.95)	
**39.3-69.8**	2.81 (1.14 , 6.9)	2.43 (1.32 , 4.46)	3.19 (2.17 , 4.7)	2.92 (2.05 , 4.17)	3.74 (2.70 , 5.17)	

*Adjusted for age, sex, ethnicity and baseline measures of smoking, alcohol consumption, diabetes status physical activity and deprivation HGS – hand grip strength

Table S13. Adjusted* hazard (with 95% confidence interval) ratios of all-cause and CVD mortality, and combined fatal/non CVD events by quintiles of Fat mass (%) and HGS in participants with history of CVD.

		All-cause mortality					
		HGS Quintile (kg)					
		43-90	35-42	29-34	23-28	0-22	P value for interaction
	**5-23.8**	1 (ref)	1 (0.79 , 1.27)	1.54 (1.2 , 1.96)	2.08 (1.56 , 2.79)	2.54 (1.86 , 3.46)	0.002
	**23.9-28.6**	0.9 (0.71 , 1.14)	1.13 (0.9 , 1.4)	1.13 (0.88 , 1.45)	1.41 (1.06 , 1.89)	1.75 (1.31 , 2.36)	
	**28.7-33.5**	1.12 (0.88 , 1.42)	1.09 (0.87 , 1.36)	1.2 (0.94 , 1.52)	1.74 (1.35 , 2.24)	1.93 (1.49 , 2.5)	
	**33.6-39.2**	1.36 (0.99 , 1.85)	1.37 (1.05 , 1.77)	1.43 (1.11 , 1.86)	1.6 (1.22 , 2.11)	1.57 (1.21 , 2.05)	
	**39.3-69.8**	2.14 (1.23 , 3.73)	1.15 (0.72 , 1.83)	1.3 (0.93 , 1.82)	1.55 (1.17 , 2.05)	1.87 (1.45 , 2.41)	
		Fatal/non-fatal CVD					
		HGS Quintile (kg)					
		43-90	35-42	29-34	23-28	0-22	P value for interaction
**Fat mass quintile (%)**	**5-23.8**	1 (ref)	0.98 (0.85 , 1.13)	1.05 (0.89 , 1.24)	1.05 (0.84 , 1.32)	1.06 (0.81 , 1.38)	0.333
	**23.9-28.6**	1.14 (0.99 , 1.31)	1.09 (0.95 , 1.25)	0.99 (0.84 , 1.16)	1.1 (0.9 , 1.34)	1.1 (0.89 , 1.36)	
	**28.7-33.5**	1.12 (0.97 , 1.31)	1.21 (1.05 , 1.39)	1.23 (1.06 , 1.43)	1.1 (0.92 , 1.31)	1.31 (1.1 , 1.56)	
	**33.6-39.2**	1.43 (1.17 , 1.74)	1.29 (1.08 , 1.53)	1.36 (1.15 , 1.61)	1.28 (1.07 , 1.53)	1.36 (1.15 , 1.61)	
	**39.3-69.8**	2.38 (1.66 , 3.42)	1.2 (0.89 , 1.63)	1.52 (1.25 , 1.85)	1.48 (1.24 , 1.76)	1.54 (1.31 , 1.81)	
		Fatal CVD					
		HGS Quintile (kg)					
		43-90	35-42	29-34	23-28	0-22	P value for interaction
	**5-23.8**	1 (ref)	1.01 (0.7 , 1.46)	1.58 (1.08 , 2.31)	2.55 (1.66 , 3.9)	3.23 (2.06 , 5.08)	0.004
	**23.9-28.6**	0.78 (0.53 , 1.15)	1.3 (0.93 , 1.82)	1.29 (0.89 , 1.87)	1.59 (1.03 , 2.47)	1.91 (1.21 , 3.01)	
	**28.7-33.5**	1.23 (0.85 , 1.78)	1.24 (0.88 , 1.74)	1.19 (0.82 , 1.73)	1.88 (1.27 , 2.78)	2.3 (1.54 , 3.42)	
	**33.6-39.2**	1.68 (1.07 , 2.62)	1.73 (1.18 , 2.53)	1.93 (1.32 , 2.82)	2.03 (1.34 , 3.09)	1.88 (1.24 , 2.83)	
	**39.3-69.8**	2.5 (1.13 , 5.53)	1.2 (0.59 , 2.46)	1.81 (1.1 , 3)	1.78 (1.13 , 2.81)	2.17 (1.45 , 3.27)	

*Adjusted for age, sex, ethnicity and baseline measures of smoking, alcohol consumption, diabetes status physical activity and deprivation HGS – hand grip strength

Table S14. Results of MR using continuous genetic risk scores with interaction between HGS and BMI/WHR.

	Outcome	HR per unit increase in HGS at the median BMI/WHR	HR per unit increase in BMI/WHR at the median HGS	Interaction term (HR)	Interaction p-value
WHR	Fatal/Non Fatal CVD	1.03 (0.97 , 1.08)	1.04 (1.00 , 1.07)	0.95 (0.87 , 1.03)	0.205
	CVD Mortality	0.88 (0.75 , 1.02)	0.99 (0.90 , 1.08)	0.92 (0.72 , 1.17)	0.478
	All-cause mortality	0.87 (0.80 , 0.94)	1.02 (0.96 , 1.07)	0.94 (0.82 , 1.07)	0.339

WHR – waist hip ratio, BMI – body mass index HGS – hand grip strength

Figure S1. Estimated association between body composition and fatal/non-fatal CHD and CVD events and cause specific and all-cause mortality excluding the first 2, 3 and 4 years of follow u: BMI and Hand Grip Strength.

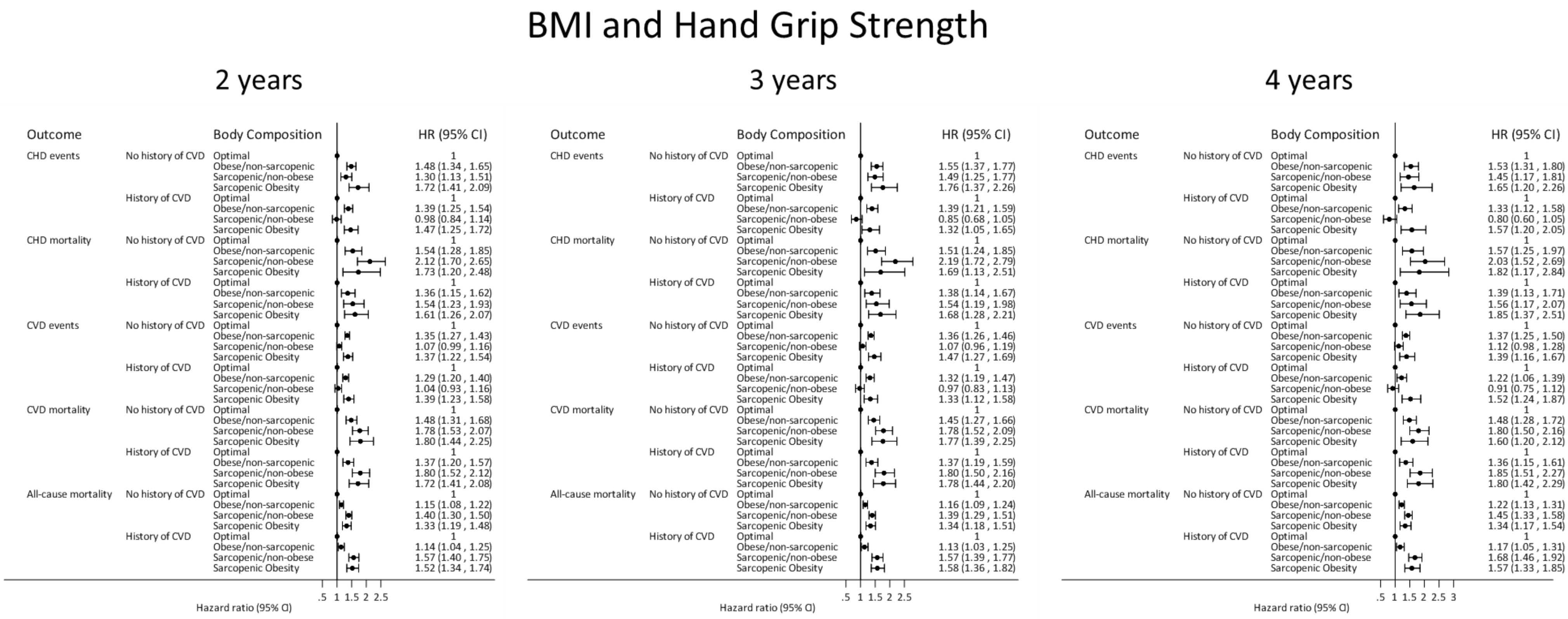

Associations (adjusted* Hazard Ratios (HRs)) estimated from a Cox Proportional Hazards model, fit separately for those with and without baseline history of CVD. Reference category is optimal body composition, i.e. not sarcopenic and not obese. Obesity measured by BMI, and sarcopenia by dominant hand grip strength (HGS).

*Adjusted for age, sex, ethnicity and baseline measures of smoking, alcohol consumption, diabetes status physical activity and deprivation

Figure S2. Estimated association between body composition and fatal/non- CHD events and mortality. Obesity measured as BMI > 30, sarcopenia measured as HGS<30kg men and <20kg women.

Outcome		Body Composition	HR (95% CI)	P-Value for Interaction
CHD events	No history of CVD	Optimal	1	0.647
		Obese/non-sarcopenic	1.45 (1.33 , 1.57)	
		Sarcopenic/non-obese	1.18 (1.06 , 1.33)	
		Sarcopenic Obesity	1.79 (1.54 , 2.08)	
	History of CVD	Optimal	1	0.598
		Obese/non-sarcopenic	1.35 (1.26 , 1.45)	
		Sarcopenic/non-obese	1.08 (0.98 , 1.20)	
		Sarcopenic Obesity	1.52 (1.37 , 1.70)	
CHD mortality	No history of CVD	Optimal	1	0.007
		Obese/non-sarcopenic	1.52 (1.28 , 1.80)	
		Sarcopenic/non-obese	1.95 (1.59 , 2.41)	
		Sarcopenic Obesity	1.74 (1.26 , 2.42)	
	History of CVD	Optimal	1	0.195
		Obese/non-sarcopenic	1.27 (1.08 , 1.49)	
		Sarcopenic/non-obese	1.47 (1.19 , 1.81)	
		Sarcopenic Obesity	1.53 (1.22 , 1.93)	

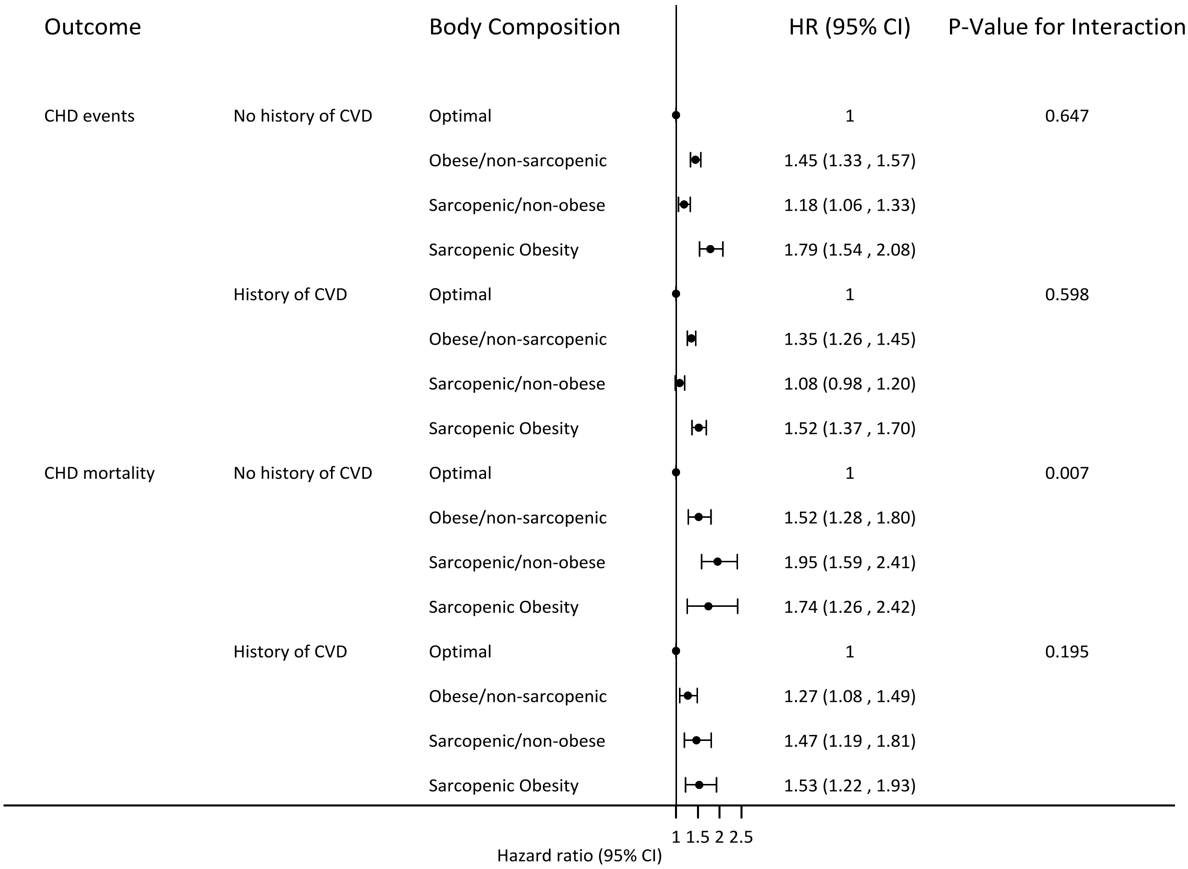

Hazard ratio (95% CI)

*Adjusted for age, sex, ethnicity and baseline measures of smoking, alcohol consumption, diabetes status physical activity and deprivation

Figure S3. Estimated association between body composition and fatal/non- CVD events and cause specific and all-cause mortality. Obesity measured as BMI>30, sarcopenia measured as SMMI in bottom 40%.

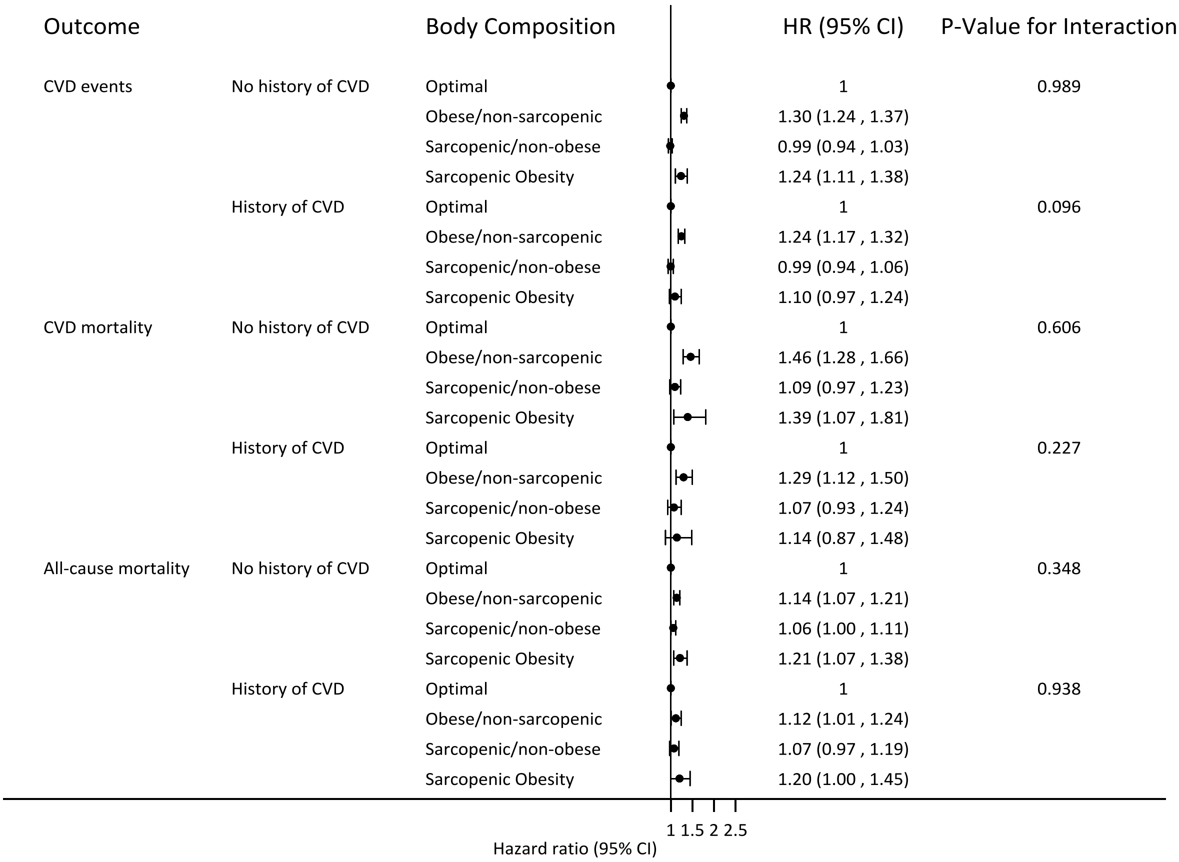

*Adjusted for age, sex, ethnicity and baseline measures of smoking, alcohol consumption, diabetes status physical activity and deprivation

Figure S4. Estimated association between body composition and fatal/non- CVD events and cause specific and all-cause mortality. Obesity measured as WHR ≥0.95 in men and ≥0.80 in women, sarcopenia measured as HGS<30kg men and <20kg women.

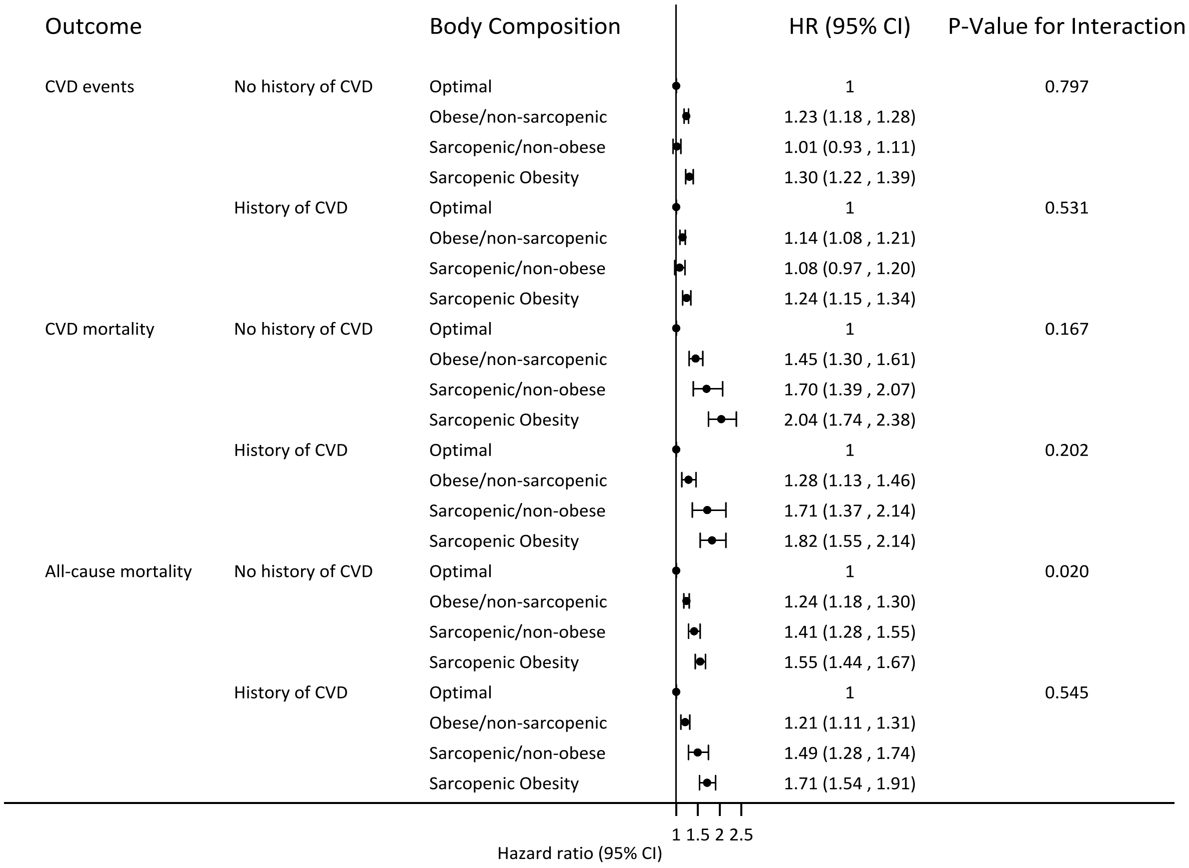

*Adjusted for age, sex, ethnicity and baseline measures of smoking, alcohol consumption, diabetes status physical activity and deprivation

Figure S5. Relative hazard of fatal/non-fatal CHD and CVD events and cause specific and all-cause mortality according to category of body composition (using WHR) as defined by genetic scores in a factorial Mendelian randomisation analysis, where weights for the genetic score were determined from both external and internal data.

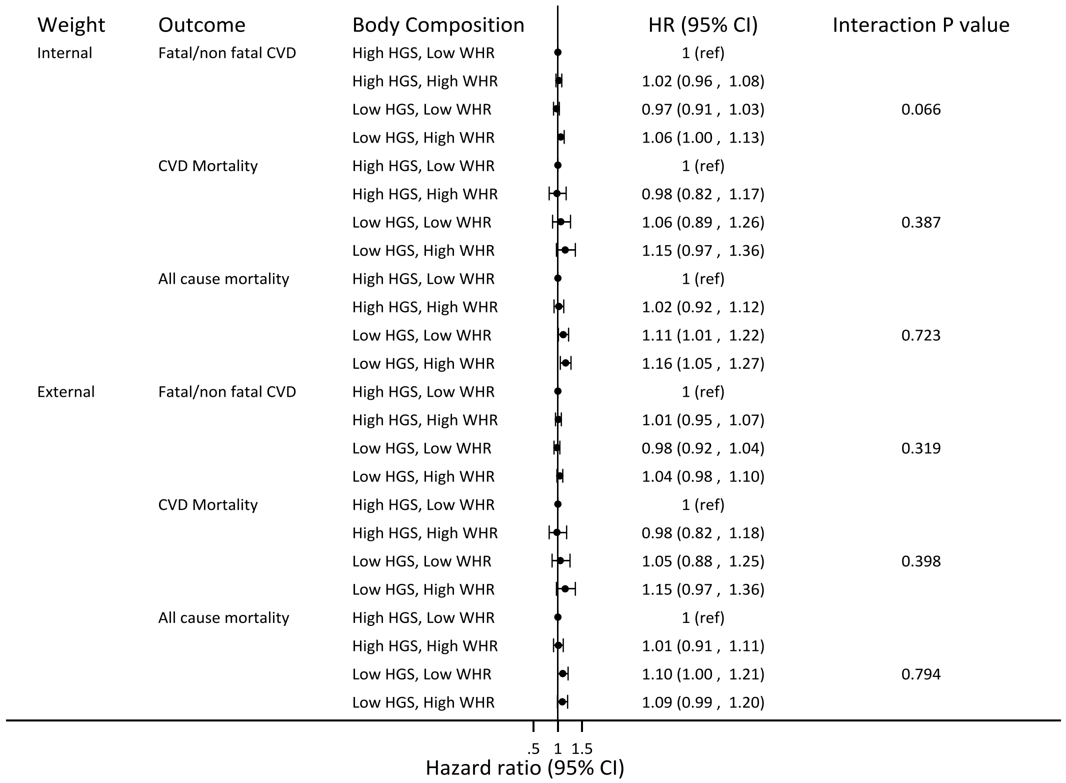

High/Low determined by cutting at the median genetic score. Associations (Hazard Ratios (HRs)) estimated from a Cox Proportional Hazards model. P value for the interaction calculated using a likelihood ratio test.

Figure S6. Relative hazard of fatal/non-fatal CHD events and mortality according to category of body composition as defined by genetic scores estimated from both external in internal weights in a factorial Mendelian randomisation analysis.

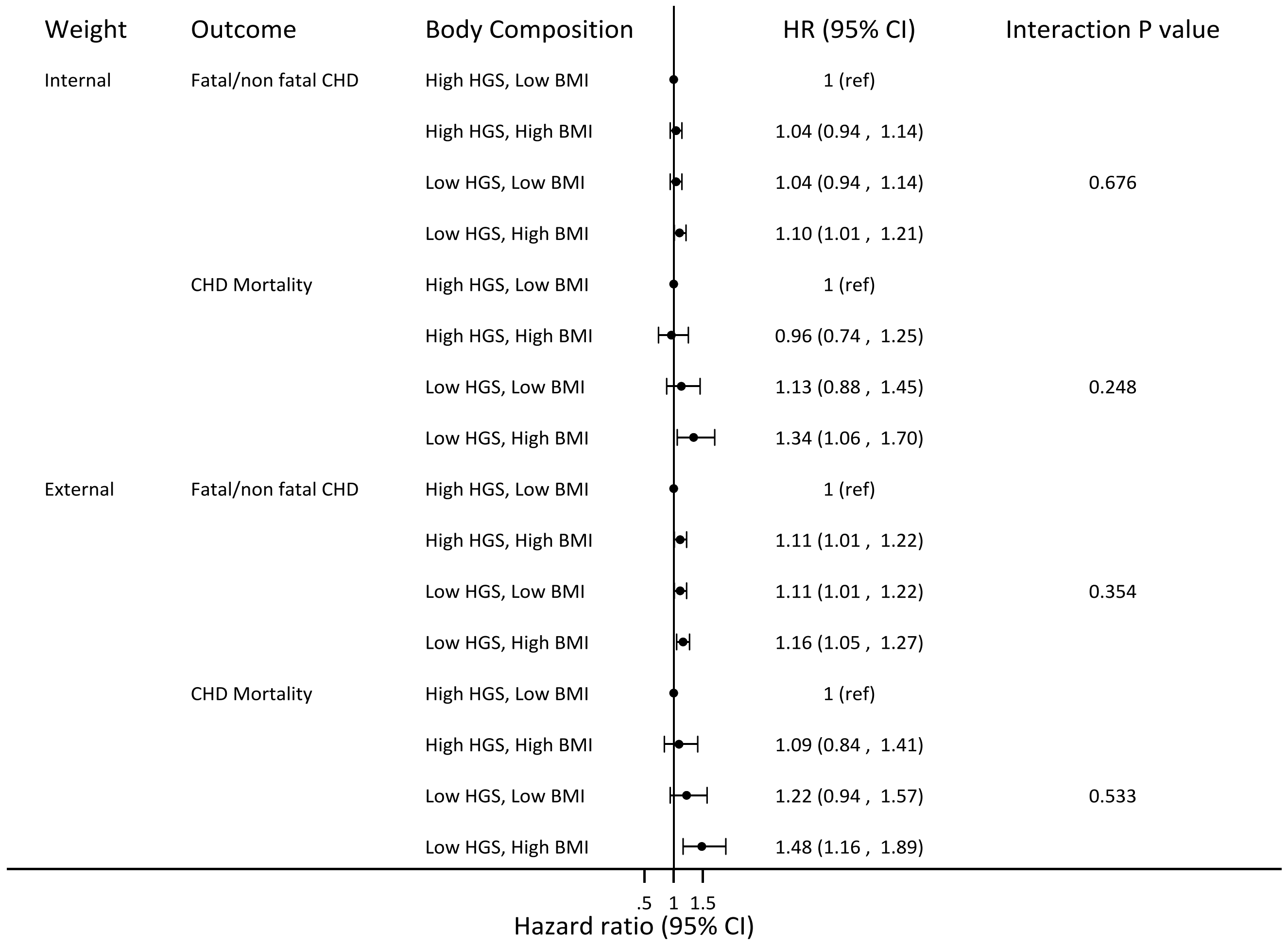

Supplement: Supplementary file 1 — Table S1. Genetic Quality Control (QC) details Table S2. Waist Hip Ratio SNPs Identified From Previous Study, and Internal/External Weights Used for MR Analyses Table S3. BMI SNPs and Internal/External Weights Used for MR Analyses Table S4. Hand‐Grip Strength SNPs and Internal/External Weights Used for MR Analyses Table S5. Adjusted* Hazard (with 95% confidence interval) Ratios of Combined Fatal/Non‐CHD Events and Mortality By Quintiles of BMI and HGS for Those Without History of CVD Table S6. Adjusted* Hazard (with 95% confidence interval) Ratios of Combined Fatal/Non‐CHD Events and Mortality By Quintiles of BMI and HGS for Those With History of CVD Table S7. Distribution of Obesity and Sarcopenia At Baseline By Sex Table S8. Adjusted* Hazard Ratios (with 95% confidence interval) of All‐Cause and CVD Mortality and Combined Fatal/Non‐CVD Events By Quintiles of BMI and SMMI in Those Without With a History of CV Table S9. Adjusted* Hazard Ratios (with 95% confidence interval) of All‐Cause and CVD Mortality and Combined Fatal/Non‐CVD Events By Quintiles of BMI and SMMI in Those With a History of CVD Table S10. Adjusted* Hazard Ratios (with 95% confidence interval) of All‐Cause and CVD Mortality and Combined Fatal/Non‐CVD Events By Quintiles of WHR and HGS in Participants Without History of CVD Table S11. Adjusted* Hazard Ratios (with 95% confidence interval) of All‐Cause and CVD Mortality and Combined Fatal/Non‐CVD Events By Quintiles of WHR and HGS in Participants With History of CVD Table S12. Adjusted* Hazard Ratios (with 95% confidence interval) of All‐Cause and CVD Mortality and Combined Fatal/Non‐CVD Events By Quintiles of Fat Mass (%) and HGS in Participants Without History of CVD Table S13. Adjusted* Hazard Ratios (with 95% confidence interval) of All‐Cause and CVD Mortality, and Combined Fatal/Non‐CVD Events By Quintiles of Fat Mass (%) and HGS in Participants With History of CVD Table S14. Results of MR Using Continuous Genetic Risk Scores With Interaction Be [file JAH3-8-e011638-s001.pdf]
